# Bromo-Substituted Phenylbenzothiazole Cyclometalating Ligands for the Development of Reverse Saturable Absorption Materials

**DOI:** 10.1021/acs.inorgchem.5c01726

**Published:** 2025-07-11

**Authors:** Erica S. Knorr, Jordan C. Kelly, Daniel P. Harrison, Catherine J. Fabiano, Caitlin G. Bresnahan, Ryan B. Gaynor, Jack M. Harrison, Nanki Verma, Trenton R. Ensley, Ryan M. O’Donnell, Kenneth J. Smith, Peter Y. Zavalij, Thomas N. Rohrabaugh, Chi K. Nguyen, Victor A. Jaffett

**Affiliations:** † 1024 U.S. Army Combat Capabilities Development Command Army Research Laboratory, 2800 Powder Mill Rd, Adelphi, Maryland 20783, United States; ‡ Department of Chemistry, 4532 Virginia Military Institute, 401 Maury-Brooke Hall, Lexington, Virginia 24450, United States; § Department of Chemistry, 3990 Rice University, 6100 Main Street, Houston, Texas 77251, United States; ∥ U.S. Army Corps of Engineers, Engineer Research and Development Center, 3909 Halls Ferry Rd, Vicksburg, Mississippi 39180, United States; ⊥ Department of Chemistry and Life Science, 8531 United States Military Academy, 753 Cullum Rd, West Point, New York 10996, United States; # Fibertek, Inc., 13605 Dulles Technology Dr, Herndon, Virginia 20171, United States; ¶ Department of Chemistry and Biochemistry, University of Maryland, 8051 Regents Drive, College Park, Maryland 20742, United States

## Abstract

Transition metal complexes have seen increased use in applications in the fields of photoredox catalysis, photodynamic therapy, biological sensing, and as phosphors for organic light-emitting diodes. Interest in transition metal complexes as nonlinear optical materials has increased as a result of advances in two-photon absorption and reverse saturable absorption (RSA) characterization techniques and observed characteristics. Iridium­(III) organometallic chromophores are of particular interest as transition metal absorbers due to their strong spin–orbit coupling, which provides access to triplet excited states and ligand-based tuning of the molecular orbitals, which enables broad spectral absorption. Herein, new microwave-assisted synthetic methods, physical characterization, and photophysical characterization of a novel series of iridium-cyclometalated with bromo-substituted phenylbenzothiazole (pbt) ligands of the form [Ir­(pbt)_2_(acac)], where acac is acetyl acetonate, are reported. At an excitation wavelength of 532 nm, all complexes demonstrate RSA behavior with the ratio of the triplet absorption cross-section to the ground state absorption cross-section (σ_T_/σ_g_) ranging from 2.8 to 7.2, the characteristics of which correlate well with electrochemical, computational, and transient absorption data.

## Graphical abstract

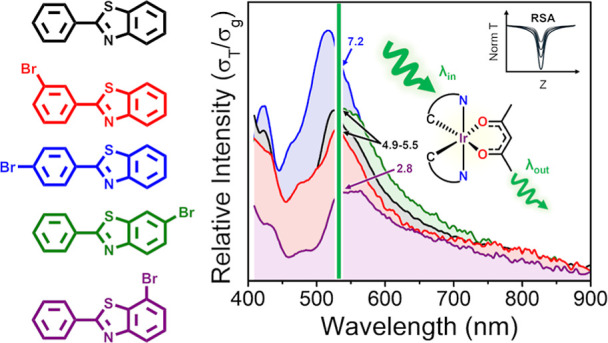

## 1 Introduction

Over the past decades, transition metal chromophores have attracted the attention of fields such as photoredox catalysis,^1,2^ photodynamic therapy,^3^ and biological sensing.^4^ The interest in these complexes stems from the large range of d-electron configurations, oxidation states, and variations in molecular structure through rational ligand design, which enable the tunability of photophysical properties,^5^ particularly because the coordinated organic ligands determine the HOMO–LUMO gap. As a class of organometallic chromophore, cyclometalated iridium­(III) complexes^6–8^ exhibit tunable electronic and photophysical properties useful for phosphorescent and electrophosphorescent applications ranging from solar cells^9–14^ to sensors,^15–18^ bioimaging,^19–21^ electrochemical cells,^22–25^ chemiluminescence,^26–29^ organic light-emitting diodes,^30–32^ and nonlinear optics (NLO).^33^ These applications are made possible by the strong spin–orbit coupling inherent to 5d^6^ iridium complexes that allow for the presence of singlet and triplet excited states accessed through efficient and ultrafast intersystem crossing.^34–40^ Additionally, Ir­(III) complexes tend to exhibit high photostability and large quantum yields.^35,36^

More recent studies demonstrated reverse saturable absorption (RSA)^41^ properties for organometallic iridium complexes with C^∧^N cyclometalating ligands and acetylacetonate (acac), [Ir^III^(C^∧^N)_2_(acac)]^0^. RSA is a phenomenon in which a material absorbs light more strongly in the excited state than in its ground state^42–46^ and is the representative mechanism for a subclass of NLO materials.

The mechanism of RSA is best described by a five-level model (Figure 1). First, a chromophore is excited to the first singlet excited state, S_1_, via the ground state linear absorption cross section, σ_g_. Depending on the chromophore and the laser excitation, two processes may occur. If the chromophore exhibits an intersystem crossing rate, *k*_ISC_, slower than the laser temporal width, then the chromophore will mostly be excited to the next singlet excited state, S_n_, through the singlet absorption cross section, σ_S_. In contrast, a chromophore having *k*_ISC_ faster than the laser temporal width will mostly intersystem cross to the lowest-lying triplet state, T_1_, followed by subsequent absorption into a higher-lying triplet state, T_*n*_, with a triplet absorption cross section, σ_T_. Previous studies have indicated that chromophores of the type being presented herein have ultrafast intersystem crossing rates (on the order of ∼100 fs),^34–40^ much faster than the ns excitation pulses used in this work for nonlinear optical interrogation. Thus, for the sake of data analysis presented herein, we neglect singlet state absorption and consider only σ_T_ contributions when determining the viability of RSA.

**Figure 1:**
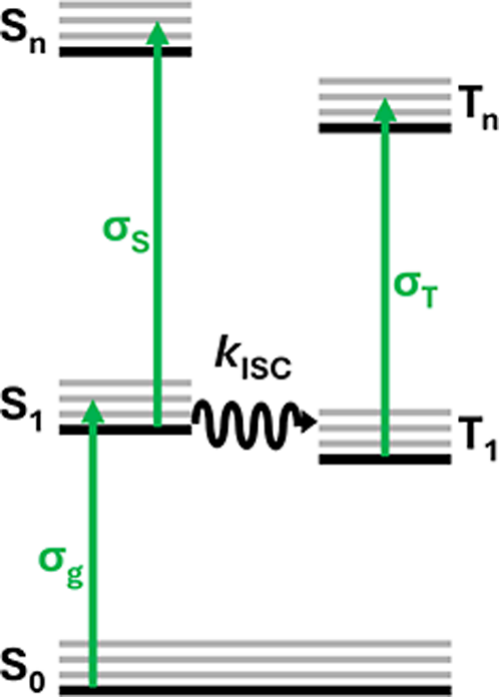
Five-level pseudo-Jablonski diagram (σ_g_ = ground state absorption cross-section; σ_S_ = singlet absorption cross-section; σ_T_ = triplet absorption cross-section; *k*_ISC_ = rate constant for intersystem crossing).

The RSA behavior depends on excited state absorption (ESA) being greater than that of the ground state; thus, the ratio, σ_T_/σ_g_, is often used to determine RSA effectiveness. This process results in an overall absorption, which increases as the fluence of incident light increases.

RSA materials have potential applications in optical and electrical switching^47–52^ as well as pulse shaping and modulation in lasers.^53–59^ Prior work demonstrated the utility of Ir­(III) cyclometalated chromophores as RSA candidates.^60–62^ Collectively, these properties make Ir­(III) complexes of interest for systematic photophysical studies to leverage their tunability.

Here, we report the synthesis of 3-, 4-, 6-, and 7-bromo-substituted 2-phenylbenzothiazole (pbt) ligands integrated into iridium cyclometalated complexes of the form [Ir­(pbt)_2_(acac)] (Figure 2, complexes **1**–**5**) and the subsequent photophysical impact of placing the bromo-substituent on either the cyclometalating ring (**2**, **3**) or the benzothiazole portion of the C^∧^N ligand (**4**, **5**). Ground state HOMO–LUMO gaps were evaluated via electrochemical analysis and computational modeling. Additionally, the photophysical properties of **1**–**5** were investigated by UV–vis absorption, emission, transient absorption (TA) spectroscopy, and Z-scan techniques to provide insight into the RSA capabilities of the new complexes.

**Figure 2:**
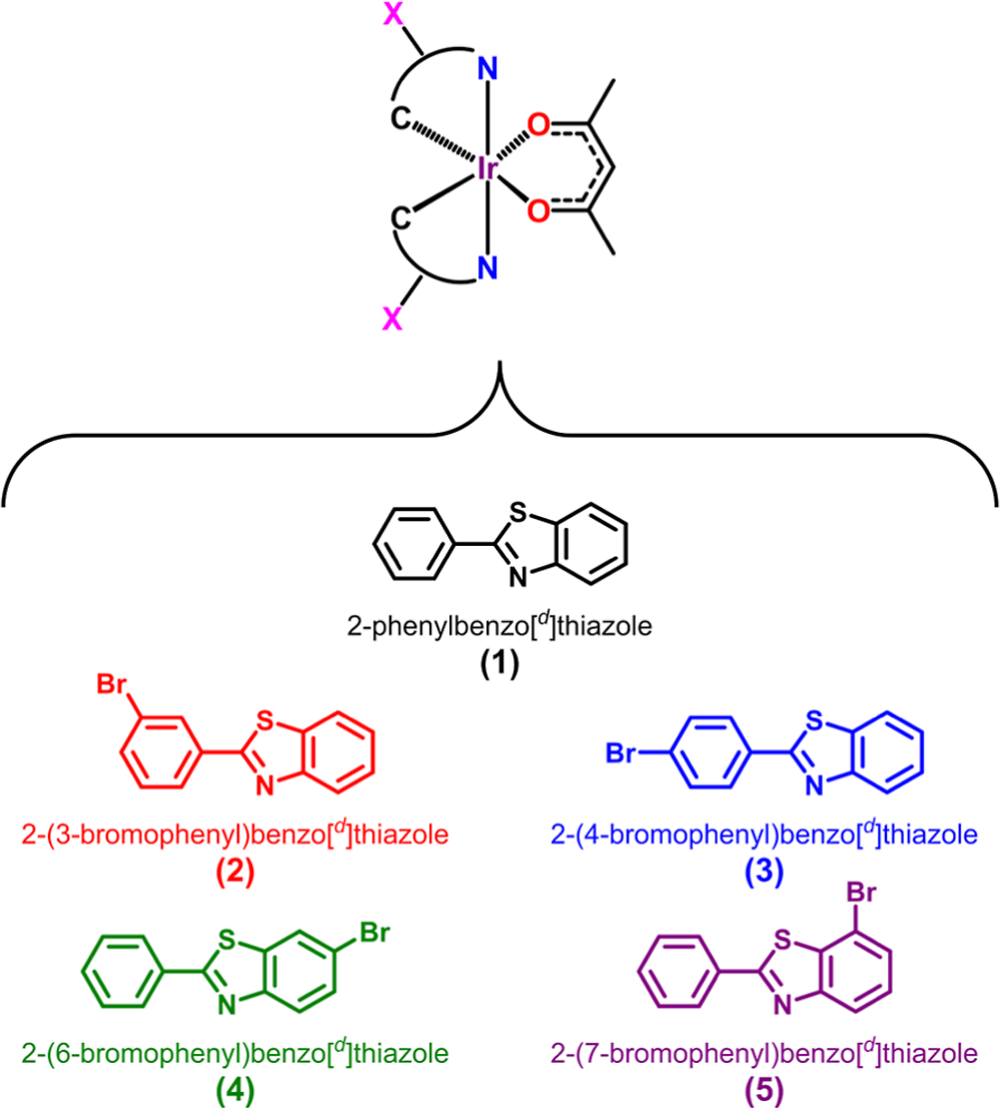
Naming scheme and colors used in this publication for bromo-substituted Ir­(III) chromophores.

## 2 Experimental Section

### 2.1 Synthesis

#### 2.1.1 Materials

All reagents and solvents were purchased from Tokyo Chemical Industry Co., Ltd. (TCI), Pressure Chemicals Co., MilliporeSigma, or CreaGen Inc. and used as received. Flash chromatography separations were carried out by using a Buchi Reveleris X2-UV or Teledyne CombiFlash NextGen 100 system with silica gel columns (normal-phase). Microwave-irradiated reactions were carried out using an Anton Paar Monowave 450 Microwave Synthesis Reactor or a CEM Discover 2.0 Microwave Reactor. All compounds were characterized with the following instrumentation: Bruker AV III HD NanoBay 400 MHz NMR or Bruker Avance III 400 MHz NMR spectrometer in CDCl_3_ (CDCl_3_: ^1^H = δ 7.26 ppm) or DMSO-*d*_6_ (DMSO: ^1^H = δ 2.50 ppm);^63^ mass spectra obtained on a JEOL AccuTOF instrument in ESI+ mode. Complex **1**, [Ir­(pbt)_2_(acac)], was synthesized via reported procedures.^64^ The synthetic details for the ligand 2-(3-bromophenyl)-benzothiazole (3-Brpbt) and iridium dimers [Ir­(3-Brpbt)_2_(μ-Cl)]_2_, [Ir­(4-Brpbt)_2_(μ-Cl)]_2_, [Ir­(6-Brpbt)_2_(μ-Cl)]_2_, and [Ir­(7-Brpbt)_2_(μ-Cl)]_2_ can be found in the Supporting Information.

#### 2.1.2 Synthesis of [Ir­(L)_2_(acac)] Complexes

##### 2.1.2.1 [Ir­(3-Brpbt)_2_(acac)] (2)

A 483 mg portion of [Ir­(3-Brpbt)_2_(μ-Cl)]_2_ (0.300 mmol) was suspended in 10 mL of dimethoxyethane in a microwave vial, followed by 0.6 mL of acetylacetone (6 mmol) and 0.6 mL of 1 M tetra-*N*-butylammonium hydroxide in methanol (0.6 mmol), and capped. The mixture was loaded into the microwave reactor and heated to 130 °C for 1.5 h. After completion, 100 mL of water was added, and the resulting precipitate was collected by vacuum filtration and washed with water, ethanol, and hexanes. The filter cake was dissolved in dichloromethane and filtered to remove insoluble particles. The filtrate was collected, and the solvent was removed by rotary evaporation, resulting in 478 mg (0.550 mmol, 91.7%) of the orange product. ^1^H NMR (400 MHz, DMSO-*d*_6_): δ 8.32–8.27 (m, 2H), 7.98 (d, *J* = 2.1 Hz, 2H), 7.94–7.89 (m, 2H), 7.62–7.54 (m, 4H), 6.81 (dd, *J* = 8.2, 2.2 Hz, 2H), 6.13 (d, *J* = 8.3 Hz, 2H), 5.20 (s, 1H), 1.72 (s, 6H). ESI-MS: [M-acac+2CH_3_CN]^+^ experimental = 852.90 *m*/*z*, calculated = 852.91 *m*/*z*. Crystals suitable for single-crystal X-ray crystallography were grown by layering ethanol on top of a concentrated solution of **2** in dichloromethane and allowing the layers to mix slowly at room temperature.

##### 2.1.2.2 [Ir­(4-Brpbt)_2_(acac)] (3)

483 mg of [Ir­(4-Brpbt)_2_(μ-Cl)]_2_ (0.300 mmol) was suspended in 10 mL of dimethoxyethane in a microwave vial, followed by 0.6 mL of acetylacetone (6 mmol) and 0.6 mL of 1 M tetra-*N*-butylammonium hydroxide in methanol (0.6 mmol), and capped. The mixture was loaded into the microwave reactor and heated to 130 °C for 1.5 h. After completion, 100 mL of water was added, and the resulting precipitate was collected by vacuum filtration and washed with water, ethanol, and hexanes. The filter cake was dissolved in dichloromethane and filtered to remove insoluble particles. The filtrate was collected, and the solvent was removed by rotary evaporation, resulting in 485 mg (0.557 mmol, 92.9%) of orange product. ^1^H NMR (400 MHz, DMSO-*d*_6_): δ 8.33–8.28 (m, 2H), 7.94–7.88 (m, 2H), 7.78 (d, *J* = 8.2 Hz, 2H), 7.60 (tt, *J* = 7.2, 5.5 Hz, 4H), 7.12 (d, *J* = 10.1 Hz, 2H), 6.26 (d, *J* = 2.0 Hz, 2H), 5.22 (s, 1H), 1.74 (s, 6H). ESI-MS: [M-acac+2CH_3_CN]^+^ experimental = 852.88 *m*/*z*, calculated = 852.91 *m*/*z*. Crystals suitable for single-crystal X-ray crystallography were grown by layering ethanol on top of a concentrated solution of **3** in dichloromethane and allowing the layers to mix slowly at room temperature.

##### 2.1.2.3 [Ir­(6-Brpbt)_2_(acac)] (4)

484 mg of [Ir­(6-Brpbt)_2_(μ-Cl)]_2_ (0.300 mmol) was suspended in 10 mL of dimethoxyethane in a microwave vial, followed by 0.6 mL of acetylacetone (6 mmol) and 0.6 mL of 1 M tetra-*N*-butylammonium hydroxide in methanol (0.6 mmol), and capped. The mixture was loaded into the microwave reactor and heated to 130 °C for 1.5 h. After completion, 100 mL of water was added, and the resulting precipitate was collected by vacuum filtration and washed with water, ethanol, and hexanes. The filter cake was dissolved in dichloromethane and filtered to remove insoluble particles. The filtrate was collected, and the solvent was removed by rotary evaporation, resulting in 517 mg (0.594 mmol, 98.9%) of orange product. ^1^H NMR (400 MHz, DMSO-*d*_6_): δ 8.56 (d, *J* = 1.9 Hz, 2H), 7.84–7.74 (m, 6H), 6.87 (td, *J* = 7.6, 0.9 Hz, 2H), 6.64 (td, *J* = 7.6, 1.3 Hz, 2H), 6.23 (d, *J* = 7.4 Hz, 2H), 5.20 (s, 1H), 1.72 (s, 6H). ESI-MS: [M-acac+2CH_3_CN]^+^ experimental = 852.87 *m*/*z*, calculated = 852.91 *m*/*z*. Crystals suitable for single-crystal X-ray crystallography were grown by layering pentane on top of a concentrated solution of **4** in toluene and allowing the layers to mix slowly at room temperature.

##### 2.1.2.4 [Ir­(7-Brpbt)_2_(acac)] (5)

484 mg of [Ir­(6-Brpbt)_2_(μ-Cl)]_2_ (0.300 mmol) was suspended in 10 mL of dimethoxyethane in a microwave vial, followed by 0.6 mL of acetylacetone (6 mmol) and 0.6 mL of 1 M tetra-*N*-butylammonium hydroxide in methanol (0.6 mmol), and capped. The mixture was loaded into the microwave reactor and heated to 130 °C for 1.5 h. After completion, 100 mL of water was added, and the resulting precipitate was collected by vacuum filtration and washed with water, ethanol, and hexanes. The filter cake was dissolved in dichloromethane and filtered to remove insoluble particles. The filtrate was collected, and the solvent was removed by rotary evaporation, resulting in 343 mg (0.394 mmol, 65.7%) of orange product. ^1^H NMR (400 MHz, DMSO-*d*_6_): δ 7.91 (dd, *J* = 8.3, 0.9 Hz, 2H), 7.86 (dd, *J* = 7.7, 1.3 Hz, 2H), 7.81 (dd, *J* = 7.8, 0.9 Hz, 2H), 7.57 (t, *J* = 8.1 Hz, 2H), 6.89 (td, *J* = 7.5, 1.1 Hz, 2H), 6.66 (td, *J* = 7.5, 1.4 Hz, 2H), 6.27 (dd, *J* = 7.7, 1.0 Hz, 2H), 5.21 (s, 1H), 1.73 (s, 6H). ESI-MS: [M-acac+2CH_3_CN]^+^ experimental = 852.87 *m*/*z*, calculated = 852.91 *m*/*z*. Crystals suitable for single-crystal X-ray crystallography were grown by layering ethanol on top of a concentrated solution of **5** in dichloromethane and allowing the layers to mix slowly at room temperature.

### 2.2 Single-Crystal X-ray Diffraction

Suitable single crystals of complexes **1**–**3**, **5** were selected and measured on a Bruker Smart Apex II CCD diffractometer. The crystals were kept at 296(2) K during the data collection. The integral intensities were corrected for absorption using SADABS software^65^ using a multiscan method.

For complex **4**, a suitable single crystal was selected and measured on a Rigaku Corporation XtaLAB Mini II diffractometer with an Oxford Cryosystems Cryostream 800 and an accompanying Oxford AD61 Dry Air Unit. The crystal was kept at 170.0(1) K during data collection. Data collection and reduction were done using CrysAlisPro; the integral intensities were corrected for absorption using the analytical numerical method.^66^ Using Olex2 1.5,^67^ the structures were solved with ShelXT^68^ and refined with ShelXL,^69^ while least-squares minimization was conducted with ShelX.^69^

### 2.3 Electrochemical Analysis

Electrochemical measurements were conducted with a CH Instruments 760E potentiostat and referenced internally versus ferrocene.^70^ Cyclic voltammetry (CV) experiments were performed in a 3-compartment glass cell similar to a double H-cell, separated by medium or fine porosity glass frits. A 3 mm diameter glassy carbon (GC) electrode was suspended in the center compartment with a septum containing 2 predrilled holes: one for the GC electrode and one for the degassing tube. In one outer compartment, a reference electrode was suspended in a septum containing 2 predrilled holes: one for the reference electrode and one for a degassing tube. In the remaining outer compartment, a platinum wire or gauze was threaded through a septum adjacent to a degassing tube and served as the counter electrode. The center compartment served as the analysis compartment and suspended the working electrode. Prior to use, each compartment was rinsed with anhydrous tetrahydrofuran (THF) solutions containing 0.1 M tetra-*N*-butylammonium hexafluorophosphate (TBAPF_6_); the reference and counter electrode outer compartments were then filled with the electrolyte solution, and the central working compartment was filled with the iridium-electrolyte solution. The solutions were freed of oxygen by vigorously degassing the electrolyte solutions with nitrogen that flowed through a gas washer containing THF. Solution sparging was performed for a minimum of 5 min, or until oxygen was no longer electrochemically detected. The GC electrodes, purchased from CH Instruments, were polished for ∼10 s with 0.3 μm alumina polish on a polishing pad and rinsed with water followed by THF. Prior to performing electrochemical experiments, the Ag wire reference electrode solution was replaced with a stock solution of 10 mM AgNO_3_ in 0.1 M TBAPF_6_ in acetonitrile. The formal reduction potentials, $E_{1/2}^{{}^{\circ}\prime }$ , were obtained by averaging the cathodic formal peak potential, $E_{p,c}^{{}^{\circ}\prime }$ , and anodic formal peak potential $E_{p,a}^{{}^{\circ}\prime }$ , of the reversible couple. $E_{p,c}^{{}^{\circ}\prime }$ ­(*L*_*π*_^0/–1^) values were determined from the cathodic peak from the first scans. The electrochemical measurements were performed using IR compensation.

### 2.4 Computational Analysis

Crystal structures were used as a starting geometry for optimization for 1,^71^ **2**, **3**, and **5**. Complex **4** was generated by altering the location of the bromine of the crystal structure for **5**. All calculations were carried out in the Gaussian 16 suite of programs.^72^ Following a model in previous work examining similar compounds,^73,74^ structures in this work were optimized with density functional theory (DFT) in their singlet ground state using the B3LYP^75–77^ functional. A pseudopotential was applied to the core electrons of iridium. The SDD^78^ basis set was used for iridium, and 6-311G* ^79,80^ was used as the basis set for bromine, sulfur, carbon, hydrogen, oxygen, and nitrogen atoms. Grimme D3 ^73^ dispersion was applied to the system. Calculations were executed with the Polarizable Continuum Model^81–83^ for solvent, which was set to toluene (ε = 2.3741). Frequency checks were performed to ensure that structures were at local minima. Time-dependent DFT was employed using the same method, basis set, and dispersion correction.^84,85^ The number of excited states calculated was 20. Molecular orbital (MO) images were generated by using IQmol.

### 2.5 Photophysical Analysis

#### 2.5.1 Absorbance

Absorption spectra were collected with a Shimadzu 2700 UV–visible Spectrometer. Solution samples were prepared by dissolving complexes in toluene in a 1 × 1 cm quartz cuvette.

#### 2.5.2 Steady-State Emission

Steady-state emission spectra were collected on a fluorescence spectrometer (Edinburgh FS5). The samples were excited at 500 nm by using light from a 150 W Xe lamp, and the emission was detected by a visible Hamamatsu photomultiplier tube. Spectra were processed with emission correction files on the Edinburgh Fluoracle software package (v. 2.9.0.4). Room temperature solution samples were prepared in freeze–pump–thaw degassed toluene in a 1 × 1 cm quartz cuvette. Emission spectra were collected in triplicate with a 1 nm step size and a dwell time of 0.5 s. Samples at 77 K were prepared in 2-MeTHF in an NMR tube and placed into a liquid nitrogen dewar. Twenty emission spectra were collected and averaged for each sample with a step size of 1 nm and a dwell time of 0.1 s.

#### 2.5.3 Franck–Condon Line Shape Analysis (FCLSA)

FCLSA was performed using the ARLSpectralFitting software (v.1.0).^86^ Room temperature spectra were fit with the single-point eq (eq 1) and 77 K spectra were fit with the double-point eq (eq 2).

1 $$I\left(\mathrm{\nu ̅}\right)=\sum_{\nu =0}^{N}\left[{\left(\frac{E_{0}-\nu \hbar \omega }{E_{0}}\right)}^{3}\left(\frac{S^{\nu }}{\nu !}\right)\times \exp\left(-4\ln\left(2\right){\left(\frac{\mathrm{\nu ̅}-E_{0}+\nu \hbar \omega }{\Delta \nu_{1/2}}\right)}^{2}\right)\right]$$

2 $$I\left(\mathrm{\nu ̅}\right)=\sum_{\nu_{M}=0}^{N}\sum_{\nu_{L}=0}^{N}\left[{\left(\frac{E_{0}-\nu_{M}\hbar \omega_{M}-\nu_{L}\hbar \omega_{L}}{E_{0}}\right)}^{4}\left(\frac{S^{\nu_{M}}}{\nu_{M}!}\right)\left(\frac{S^{\nu_{L}}}{\nu_{L}!}\right)\times \exp\left(-4\ln\left(2\right){\left(\frac{\mathrm{\nu ̅}-E_{0}+\nu_{M}\hbar \omega_{M}+\nu_{L}\hbar \omega_{L}}{\Delta \nu_{1/2}}\right)}^{2}\right)\right]$$

In these equations, *I* = intensity as a function of wavenumber (ν̅); *N* = number of quantum levels; ν = vibronic quantum number; *E*_0_ = 0–0 energy gap; Δν_1/2_ = full width at half-maximum; ℏω = quantum spacing parameter; and *S* = Huang–Rhys factor. *M* and *L* represent medium- and low-frequency vibrational modes, respectively.^86–89^

#### 2.5.4 Quantum Yield

Emission quantum yields of complexes in freeze–pump–thaw degassed toluene were measured relative to a standard of Rhodamine 6G in ethanol (ϕ_*f*_ = 0.95).^90^ Absorbance values at 500 nm were prepared to 0.05–0.08 O.D. Samples were excited at 500 nm with a slit width of 2.25 nm, and emission was collected with a slit width of 1 nm. Three separate photoluminescence spectra were collected from 510 to 900 nm (step size 1 nm, dwell time 0.5 s) for each sample. The integrated emission intensity was calculated using the Edinburgh Fluoracle software package (v. 2.9.0.4). Relative quantum yields were calculated using eq 3.^91,92^

3 $$\phi_{S}=\phi_{R}\left(\frac{I_{S}}{I_{R}}\right)\left(\frac{1-10^{-A_{R}}}{1-10^{-A_{S}}}\right){\left(\frac{\eta_{S}}{\eta_{R}}\right)}^{2}$$

where *S* = sample; *R* = reference; ϕ = quantum yield; *I* = integrated emission intensity (510–900 nm); *A* = absorbance at 500 nm; and η = refractive index of the solvent.

#### 2.5.5 Time-Resolved Emission

Time-resolved emission measurements were recorded at room temperature on the same fluorescence spectrometer. The samples were excited by a pulsed diode laser (operating at 510 ± 10 nm, having a pulse width of 85 ps) with a repetition rate of 50 kHz (Edinburgh EPL-510 nm). Emission decay traces were acquired using time-correlated single photon counting (TCSPC; 1024 channels; 20 μs window) with data collection for 5000 counts. The decay traces were fit with a monoexponential tail fit using the Edinburgh Fluoracle software package (v. 2.9.0.4).

#### 2.5.6 Transient Absorption (TA)

TA measurements were collected using a quasi-collinear pump–probe geometry, in which the pump and probe beams incident on the sample are separated by a small angle (<10°), in a commercial TA spectrometer (Ultrafast Systems EOS) with aerated samples in 1 mm cuvettes. The 400 nm pump beam was generated by frequency doubling the 800 nm output from an amplified Ti/sapphire femtosecond laser system (Spectra-Physics Solstice), producing 3.5 mJ, 100 fs (full width at half-maximum, fwhm) pulses at a 1 kHz repetition rate. A photonic crystal fiber is used to generate the white-light continuum probe pulse (350–950 nm, 0.5 ns pulse width, 20 kHz repetition rate) that is digitally synchronized with the 400 nm pump beam. The probe is split into a reference and sample line and then coupled into its own fiber-optic spectrometer. All TA data were corrected by subtracting out spectral differences of the sample line from the reference line.

#### 2.5.7 Z-Scans

To determine triplet state cross sections, the conventional Z-scan technique was utilized.^93^ Single-beam Z-scans (Figure 3) were performed using an Nd/YAG nanosecond laser source (Ekspla, NL120) that produced 532 nm, 2 mJ, and 7 ns fwhm pulses at a 10 Hz repetition rate. The laser beam passed through an all-reflective spatial filter before being directed to the Z-scan line. After the spatial filter, a BK7 wedge was used as a pickoff to monitor the pulse energy on a reference photodetector (Thorlabs, PDA100A2). The pulse energy was adjusted by using a half-wave plate/polarizer pair. The pulse energy was measured with a calibrated energy sensor (Ophir, PD10), and the average reading was set to the average voltage of the reference detector and set with a ±5% energy window. The minimum beam waist at the focus was determined to be 14.5 μm (half-width 1/*e* ^2^) by taking Z-scans of reference materials with known parameters. Four Z-scans were collected per sample at varying pulse energies.

**Figure 3:**
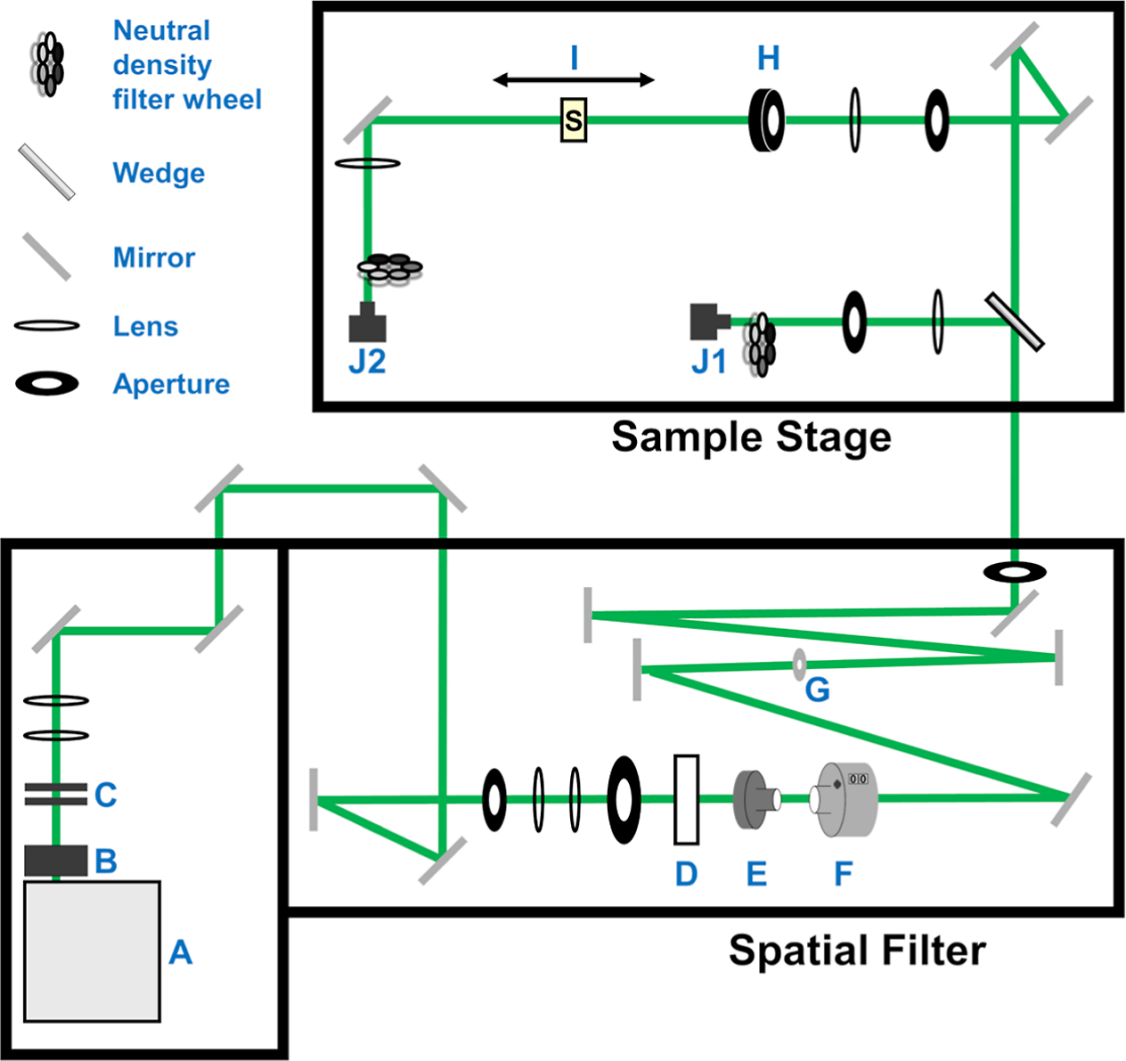
Z-scan configuration. Schematic not drawn to scale (A—laser source; B—interlocked safety shutter; C—neutral density filters; D—motorized rotation stage; E—Glan-Taylor calcite polarizer; F—neutral density filters; G—diamond pinhole; H—removable power meter; I—Z-stage; J1—reference detector; and J2—open aperture detector).

Samples were prepared in 1 mm glass cuvettes. Solutions were made by dissolving complexes **1**–**5** in toluene and adjusting the concentration to an absorbance of 0.07 O.D. at 532 nm. Solutions were filtered via a syringe filter (13 mm, 0.2 μm PTFE) before measurements. Silicon naphthalocyanine (SiNc) and C60 standards dissolved in toluene were run in addition to complexes **1**–**5** to verify laser parameters. Extensive studies have previously characterized the excited state dynamics and nonlinear optical properties of both SiNc and C60.^94–100^ A five-level pseudo-Jablonski diagram (Figure 1) is often used to describe the excited state dynamics of complexes. Because the relevant fitting parameters have been well characterized (i.e., ground, singlet, and triplet absorption cross sections; intersystem crossing yield and rate constant; and singlet and triplet decay times), SiNc and C60 are ideal reference materials. Example Z-scans of SiNc and C60 can be found in Figure S22 and Table S4.

## 3 Results

### 3.1 Synthesis and Physical Characterization

The synthetic pathway for complexes **1**–**5** modified a previously reported process (Scheme 1).^64^ First the iridium dimers, [Ir­(C^N)_2_(μ-Cl)]_2_ (where N = pbt, 3-Brpbt, 4-Brpbt, 6-Brpbt, and 7-Brpbt), were prepared by reacting 2 equiv of ligand with iridium­(III) chloride trihydrate in a 2-methoxyethanol/water (3:1, v/v) mixture aided by microwave irradiation. The resulting iridium dimers were then reacted with excess acetylacetone and tetra-*N*-butylammonium hydroxide (TBAOH) (1 M in methanol) in dimethoxymethane and subjected to microwave irradiation. Following purification, complexes **1**–**5** were obtained in moderate overall yields ranging from 62% to 91%, with structure and purity confirmed by ^1^H NMR (Figures S2–S5), ESI-MS (Figure S6), and X-ray crystallography (Figure 4).

**Scheme 1:**
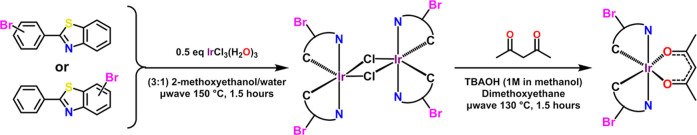
Synthesis of Bromo-Substituted Ir­(III) Cyclometalated Complexes

**Figure 4:**
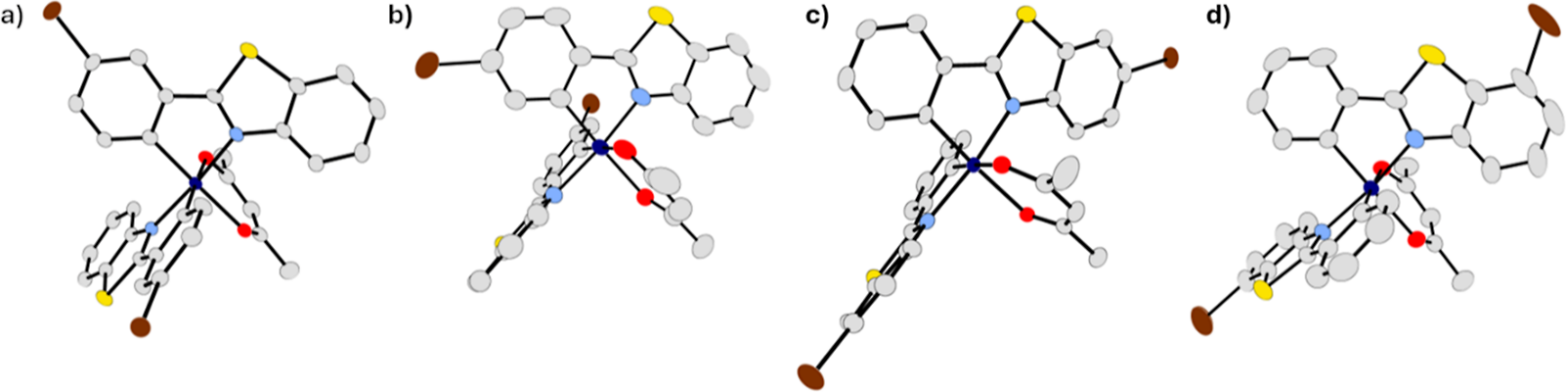
Thermal ellipsoid representations of (a) **2**; (b) **3**; (c) **4**; and (d) **5** at 40% probability. Hydrogen atoms and cocrystallized solvent molecules are omitted for clarity.

The single-crystal X-ray diffraction structures solved for **1**–**5** can be found in Figure 4, with select bond lengths and angles shown in Table 1. Full crystallographic information is provided in the Supporting Information. The complexes possess a distorted octahedral geometry with the iridium­(III) center coordinated to two pbt ligands in the typical *cis*-C,C/*trans*-*N*,*N* isomeric form with incorporation of acac as the auxiliary ligand.^71^ Iridium–carbon and iridium–nitrogen bond lengths ranged from 1.994(4) to 2.011(3) Å and 2.046(3) to 2.064(2) Å, respectively. Values are in good agreement with the parent pbt compound [Ir–C: 1.988(5) and 1.992(5) Å, Ir–N: 2.052(4) and 2.055(4) Å]. The iridium–oxygen bond lengths were characteristic for these types of complexes, ranging from 2.128(2) to 2.162(2) Å. Bite angles for the pbt ligands were nearly identical in each compound [80.2(1)-80.7(1)°] and were expectedly smaller compared to the acac ligands, which ranged from 87.03(8) to 88.29(7)°. Worth noting, the iridium–oxygen bonds are statistically shorter when the bromo is in the 3- or 4-position, possibly a consequence of varying amounts of back bonding from the iridium to the pbt ligand throughout the series. The other bond lengths and angles were found to be statistically similar, indicating minimal impact of the bromo-substitution position on the structural features.

**Table 1:** Select Average Bond Lengths (Å) and Angles (°) for **2**–**5** ^a^

complex	Ir–C Bond	Ir–N Bond	Ir–O Bond	Br–C Bond	C–Ir–N	O–Ir–O
**1**	1.990(7)	2.054(6)	2.137(4)		80.2(3)	88.9(1)
**2**	1.997(4)	2.061(3)	2.141(3)	1.897(4)	80.6(1)	88.29(7)
**3**	1.997(6)	2.052(4)	2.146(4)	1.900(5)	80.4(1)	87.4(1)
**4**	2.008(4)	2.053(4)	2.157(3)	1.910(6)	80.6(1)	88.12(9)
**5**	2.002(4)	2.057(4)	2.160(3)	1.896(6)	80.4(1)	87.03(8)

a Average bond lengths and angles of **1** are tabulated for comparison.^71^

### 3.2 Electrochemistry

To further explore how the position of bromine substitution affects molecular properties, CV was performed on the series in anhydrous THF supported by TBAPF_6_ at a 100 mV/s scan rate (Figure 5). Table 2 provides the electrochemical measurements for the complexes. All brominated compounds’ electrochemical events were anodically shifted as compared to the parent complex. All Ir^IV/III^ couples were electrochemically reversible and chemically reversible with **3** most anodically shifted (*E*°′(Ir^IV/III^, **3**) = 0.74 V) and the parent complex was the most electron rich, *E*°′(Ir^IV/III^, **1**) = 0.55 V, such that the reduction potentials of the series spanned Δ*E*°′ = 0.19 V (Δ*E*°′(Ir^IV/III^) = *E*°′(Ir^IV/III^, **3**) – *E*°′(Ir^IV/III^, **1**)). The ligand-based reduction, *L*_π_^0/–1^, was reversible for the parent complex but was irreversible for all brominated compounds. The average of the reduction wave potentials for the brominated compounds ( $E_{p,c}^{{}^{\circ}\prime }$ ­(*L*_*π*_^0/–1^) = −2.35 ± 0.02 V) was 0.09 V anodically shifted from the parent $E_{1/2}^{{}^{\circ}\prime }$ ­(*L*_*π*_^0/–1^, **1**).

**Figure 5:**
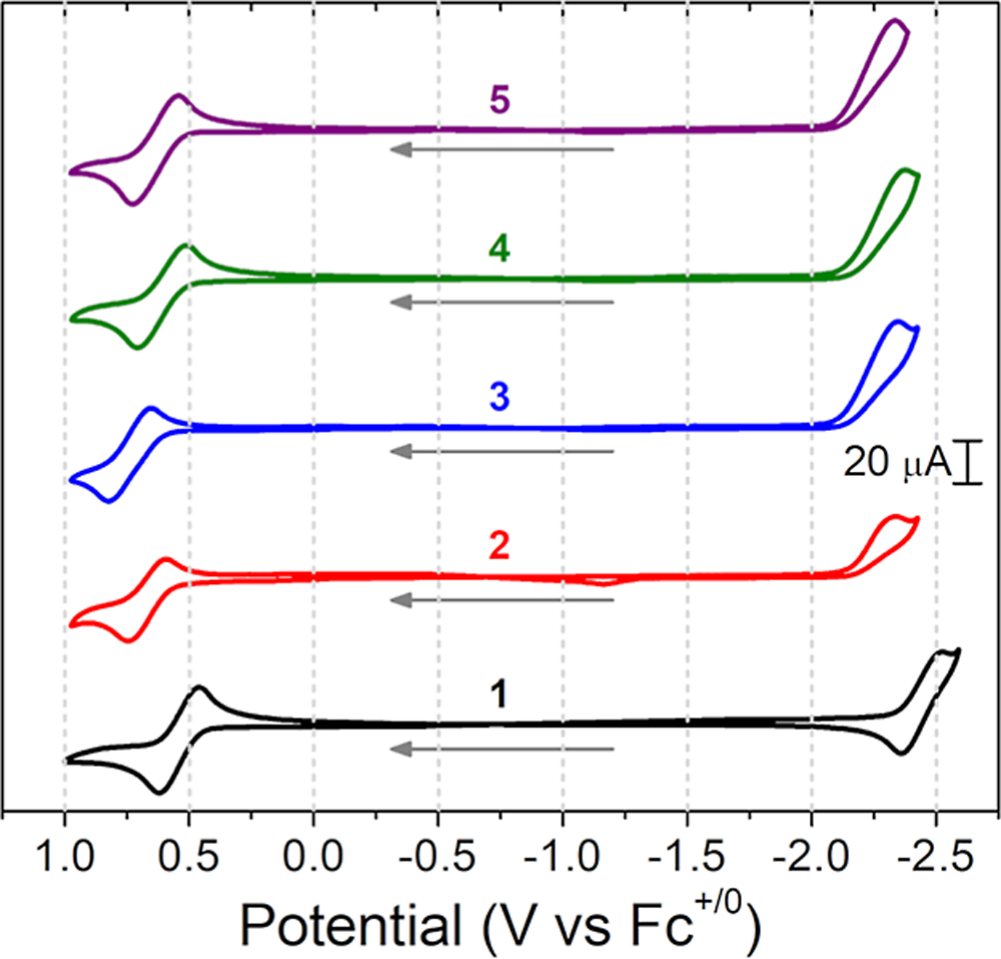
First scan cyclic voltammograms, CVs, of the title complexes collected at a 100 mV/s scan rate, in anhydrous THF containing 0.1 M TBAPF_6_ using IR compensation. The cyclic voltammograms, CVs, were collected by scanning anodically from an initial potential of −1.1 V (vs Fc^+/0^) to a switching potential of ∼1.0 V, which was followed by a cathodic scan to a switching potential 0.05–0.1 V beyond the $E_{p,c}^{{}^{\circ}\prime }$ (and *E*°′(*L*_*π*_^0/–1^) for **1**) of the complexes, and then back to −1.1 V. Gray arrows indicate initial scan direction. Traces: **1** (1.9 mM, black), **2** (1.9 mM, red), **3** (1.8 mM, blue), **4** (1.8 mM, green), and **5** (2.0 mM, purple).

**Table 2:** Measured Electrochemical Potentials for the Identified Redox Couple Measured in THF With 0.1 M TBAPF_6_ Supporting Electrolyte at 100 mV/s Scan Rate^a^

complex	*E*°′(Ir^IV/III^)/V (Δ $E_{p}^{{}^{\circ}\prime }$ /mV)	$E_{p,c}^{{}^{\circ}\prime }$ (*L*_π_^0/–1^)/V	Δ*E*_HOMO–LUMO_ ^d^/V
**1**	0.55 (159)	–2.44 (159)^b^ ^,^ ^c^	2.99
**2**	0.67 (154)	–2.34	3.01
**3**	0.74 (166)	–2.35	3.08
**4**	0.60 (185)	–2.38	2.98
**5**	0.65 (157)	–2.34	2.98

a For reversible couples, the peak-to-peak separation (Δ*E*_*p*_ $E_{p}^{{}^{\circ}\prime }$ = $E_{p,a}^{{}^{\circ}\prime }$ – $E_{p,c}^{{}^{\circ}\prime })$ of the anodic and cathodic waves is indicated in parentheses.

b Reduction of the parent compound is reversible.

c *E*°′(*L*_*π*_^0/–1^) for 1 in parentheses.

d Ground state HOMO–LUMO gap.

Scanning through the irreversible ligand-based reduction waves of the bromo-substituted complexes generated new anodic oxidation(s) upon return to the Ir^IV/III^ couple (Figure S7). For example, a new oxidation at +0.28 V was generated on an anodic scan following a cathodic scan more negative than the irreversible reduction ( $E_{p,c}^{{}^{\circ}\prime }$ ­(*L*_*π*_^0/–1^) = −2.34 V vs Fc^+/0^) of **5** (Figure 6). The new oxidation was not present when multiple anodic cycles were performed for any of the complexes (Figures 6b and S8b). The new $E_{p,a}^{{}^{\circ}\prime }$ = 0.28 V is consistent with CV data previously reported for the oxidation of bromide to tribromide in acetonitrile.^101^ Presumably, ligand reduction induces the weak C–Br bond to cleave, producing bromide (and other decomposition products), which oxidizes to Br_3_^–^ upon anodic scanning. Similar reductive cleavage has been postulated for bromo-substituted terpyridine ligands.^102^

**Figure 6:**
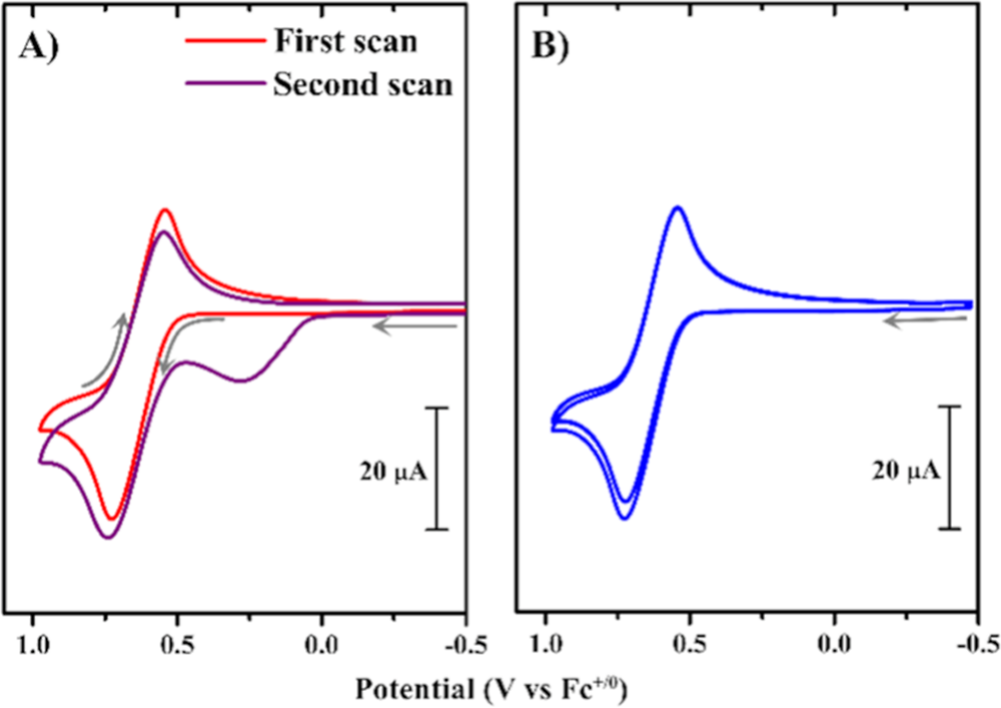
CVs of 2.0 mM 5 collected at 100 mV/s scan rate in THF containing 0.1 M TBAPF_6_. (a) Red trace indicates the first anodic scan, while the purple trace indicates the return scan after cycling through the irreversible ligand-based couple at −2.34 V (with a switching potential = −2.39 V); (b) three CV cycles (not scanning through the reduction wave) of the same 2.0 mM 5, with the cathodic switching potential of −0.475 V, collected at a 100 mV/s scan rate in THF containing 0.1 M TBAPF_6_. Gray arrows indicate scan direction.

The electrochemically measured HOMO–LUMO gap (Δ*E*_HOMO–LUMO_ = Δ*E*°′(Ir^IV/III^) – $E_{p,c}^{{}^{\circ}\prime }$ ­(L^0/–1^)) for the complexes is also presented in Table 2. Notably, 3 has the largest gap, Δ*E*_HOMO–LUMO_ = 3.08 V, while the other bromides resemble that of the parent complex. Given that the ligand-centered reduction is roughly constant among the brominated series, the Δ*E*_HOMO–LUMO_ is necessarily and primarily controlled by HOMO coupling to the bromine substituent.

### 3.3 Computational

To better explain the HOMO/LUMO deviations observed via electrochemical measurements, electron densities of the HOMO and LUMO for each complex were calculated, as described in Section 2.4, and relevant MOs are presented in Figure 7. The LUMOs of **1**–**5** are primarily ligand-based and do not significantly vary with the position of the bromine atom. The HOMOs, on the other hand, appear to be a mix of cyclometalating and iridium­(III) character. Additionally, there is greater variance in the HOMO energy levels than in the LUMO, which also agrees with the observation that the HOMO–LUMO gap is determined by HOMO coupling to the bromine. In all cases, DFT modeling indicates that the lowest energy pair of transitions are mainly composed of HOMO and HOMO–1 to LUMO and LUMO+1 transitions.

**Figure 7:**
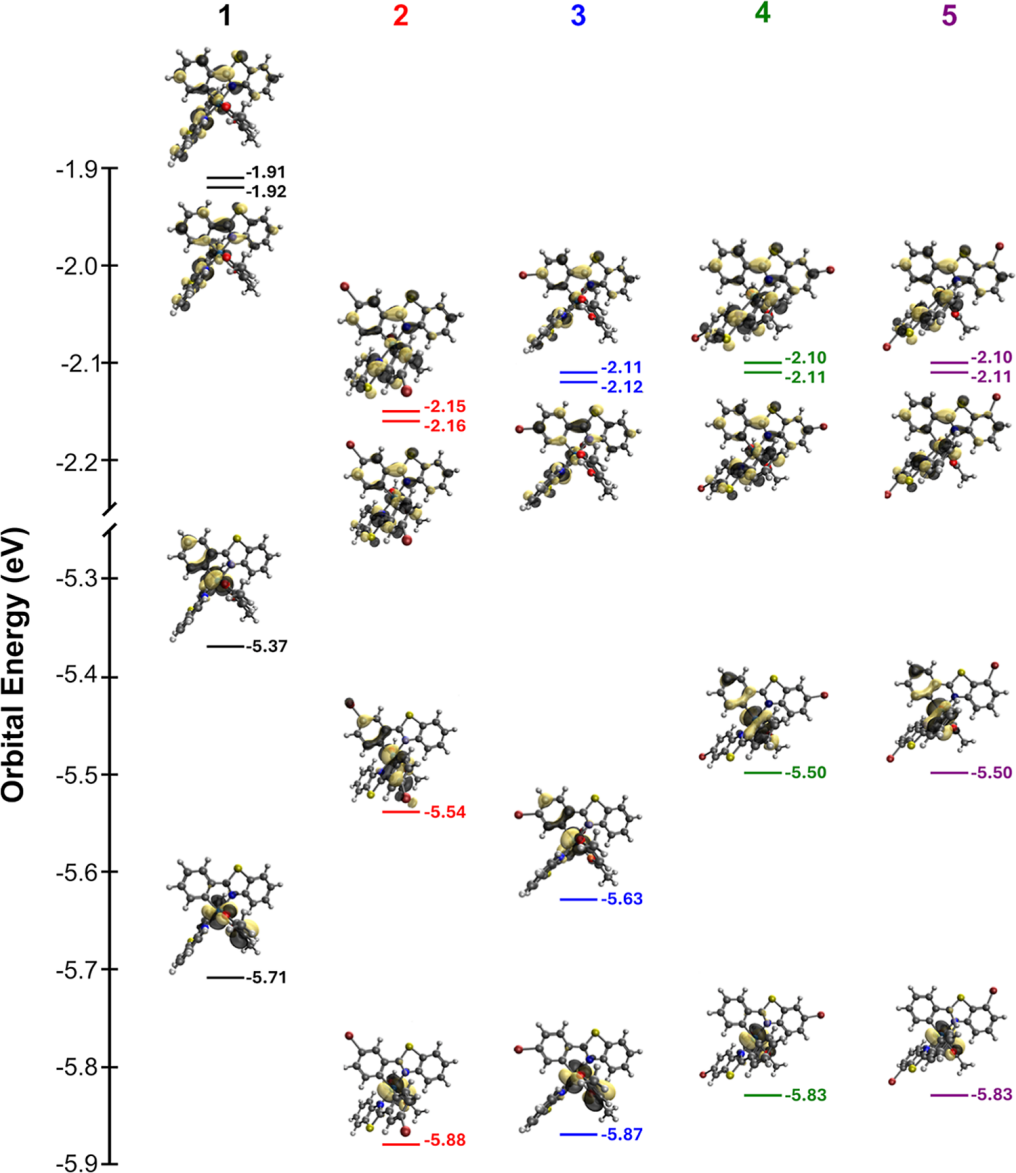
Computed MOs for the HOMO–1, HOMO, LUMO, and LUMO+1 of complexes **1**–**5**.

### 3.4 Photophysical and Nonlinear Optical Properties

The differences observed via electrochemistry and DFT calculations are manifested as photophysical changes as well. All chromophores exhibited absorption features spanning most of the visible range from 300 to 575 nm (Figure 8a,b and Table 4). The higher intensity bands from 300 to 350 nm can be attributed to ligand-centered states, while the broader, lower intensity absorption bands between 450 and 575 nm can be attributed to metal-to-ligand charge transfer (MLCT) bands, likely of mixed singlet and triplet character. The highest energy MLCTs between 450 and 575 nm represent a ±0.02–0.04 eV shift relative to that of the parent complex. Compound **3** exhibits the highest energy peak (448 nm), followed by **1**, **2**, and **4**–**5**. The peaks for **4** and **5** are approximately the same (454 nm), and computational absorbance values are in good agreement with experimental values (Figure S24). Though the MLCT features are only minimally altered across the series, the incorporation of the bromine and its position on the cyclometalating ligand are reflected in the modulation of the ground state photophysics.

**Table 3:** Calculated HOMO/LUMO Energies for Complexes **1**–**5**

complex	HOMO–1/eV	HOMO/eV	LUMO/eV	LUMO+1/eV	HOMO–LUMO Gap/eV
**1**	–5.37	–5.71	–1.91	–1.92	3.45
**2**	–5.54	–5.88	–2.15	–2.16	3.38
**3**	–5.63	–5.87	–2.11	–2.12	3.51
**4**	–5.5	–5.83	–2.1	–2.11	3.39
**5**	–5.5	–5.83	–2.1	–2.11	3.39

**Figure 8:**
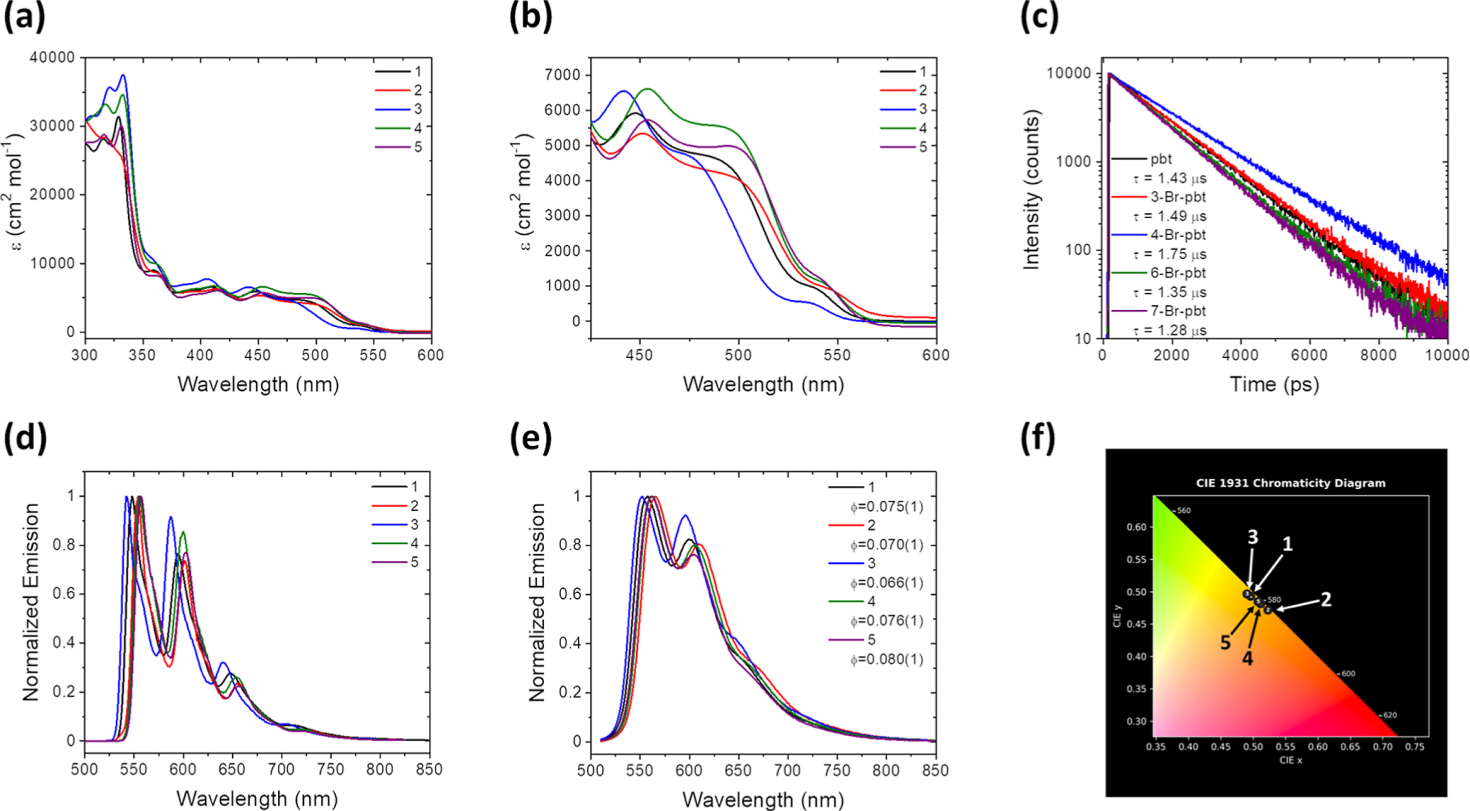
(a) UV-visible spectra in toluene; (b) absorption of the MLCT bands in toluene at room temperature; (c) emission decay traces in freeze-pump-thaw degassed toluene; (d) steady-state emission in a 2-MeTHF glass at 77 K; (e) steady-state emission in freeze-pump-thaw degassed toluene at room temperature; (f) CIE chromaticity diagram showing the color coordinates of complexes **1**–**5**.

**Table 4:** Summary of the Photophysical Properties of Complexes **1**–**5** (Em = Emission; RT = Room Temperature; *k*_nr_ = Nonradiative Decay Constant; *k*_r_ = Radiative Decay Constant; Ex = Excitation)

complex	λ_MLCT_/nm (ε/cm^2^ mol^–1^)	λ_em_ (77 K)^a^/nm	λ_em_ (RT)^b^/nm	ϕ_em_ ^b^	τ_em_ ^b^ ^,^ ^c^/μs	*k*_nr_^b^/10^5^ s^–1^	*k*_r_^b^/10^5^ s^–1^	*E*_00_^a^/eV
**1**	448 (5100)	548	558	0.75(1)	1.43	1.75	5.25	2.26
**2**	451 (5400)	554	565	0.70(1)	1.49	2.01	4.69	2.23
**3**	442 (6700)	542	552	0.66(1)	1.75	1.94	3.77	2.28
**4**	454 (6700)	555	563	0.76(1)	1.35	1.78	5.64	2.23
**5**	454 (6000)	557	562	0.80(1)	1.28	1.56	6.24	2.22

a In a 2-MeTHF glass at 77 K (λ_ex_ = 450 nm).

b In freeze-pump-thaw degassed toluene at room temperature (λ_ex_ = 500 nm).

c λ_ex_ = 510 nm.

Excited state photophysical properties of the brominated complexes were evaluated by comparing their emission spectra at 77 K and at room temperature (Figure 8d,e). At 77 K, the complexes exhibit highly structured emission with the highest energy peak falling between 543 and 557 nm. FCLSA shows that the HOMO–LUMO energy gap, *E*_00_, varies for the compounds but generally correlates well with changes in absorbance. The emission for all complexes broadens and red-shifts relative to the emission at 77 K when observed in freeze–pump–thaw degassed toluene and can be attributed to rigidochromic effects.^103,104^ The highest energy emission is observed in **3**, followed by **1**. The emission of complexes **2**, **4**, and **5** is lower than that of **1** but does not clearly trend with absorption, potentially due to peak broadening.

Comparative quantum yield measurements were also performed at room temperature using a Rhodamine 6G standard (ϕ_*f*_ = 0.95). With the exception of **2**, the quantum yield increased from 0.66 to 0.80 as the complexes’ absorption red-shifted. The rate constants for nonradiative decay (*k*_nr_) similarly decrease with increased absorption wavelength.

Time-resolved emission decays (Figure 8c) were observed via time-correlated single photon counting (TCSPC) and fit with monoexponential decay models. The excited state lifetimes for all complexes were greater than 1 μs, with **3** having the longest decay and **5** having the shortest. Monoexponential fits are consistent with one major decay process occurring on the nanosecond time scale. The most likely mechanism of emission in these Ir­(III) complexes would involve excitation to the first singlet excited state, followed by ultrafast intersystem crossing and decay from the triplet state.^34–40^ Because intersystem crossing for Ir­(III) complexes takes place within hundreds of femtoseconds, the growth of the triplet state would not be observed with a picosecond diode laser, as is used here. The significant Stokes shifts for all complexes and long-lived excited states further indicate the triplet nature of the excited state rather than the singlet excited state.

In addition to steady-state photophysics, the submicrosecond dynamics were evaluated via TA (Figure 9) in aerated toluene. All complexes show ESA features between 400 and 850 nm. The decrease in decay times obtained from the aerated samples (Table S3) is consistent with oxygen quenching in the triplet state. Additionally, while the ESA appears as two separate peaks (400–450 and 500–850 nm), the peaks decay at the same rate (Table S3, Figure S21) and therefore likely correspond to decay from the triplet state, in agreement with TCSPC measurements. The central wavelength of the lower energy ESA peak corresponds to the ground state absorption maxima of the complexes (Table S3). Though C–Br cleavage was suggested by electrochemistry, this cleavage does not appear to occur when **1**–**5** are photoexcited, as emission decays are monoexponential and TA spectra do not show evidence of photodegradation products.

**Figure 9:**
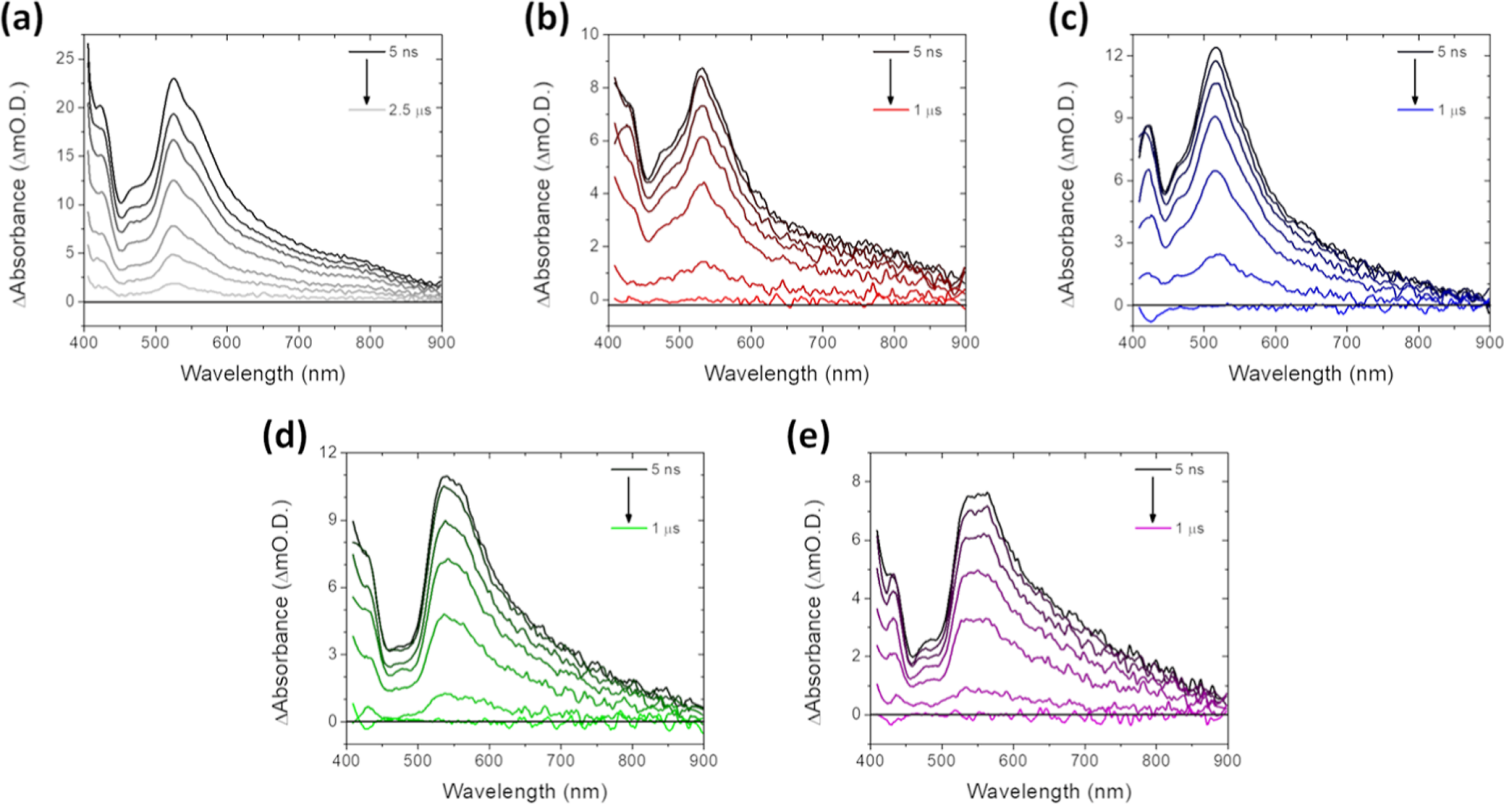
TA spectra for (a) **1**; (b) **2**; (c) **3**; (d) **4**; and (e) **5** in aerated toluene (25-point smooth).

The ESA features of complexes **1**–**5** suggest that these chromophores may be potential RSA candidates. To probe the RSA response of **1**–**5** at 532 nm, the open-aperture Z-scan technique was used with nanosecond pulses. RSA is expected to occur in spectral regions where a positive TA signal is observed and in which the positive signal overlaps with the ground state absorption. Complexes **1**–**5** exhibit positive ESA features between 400 and 900 nm in addition to ground state absorption features at 532 nm, qualitatively indicating RSA potential at 532 nm. For each sample, four Z-scans were performed with an increasing incident pulse energy. As the incident pulse energy increased, the transmittance decreased for all samples (Figures 10 and 11). These transmittance curves were subsequently fit using eqs. 1 and 2 in ref 99 with the five-level model shown figure in Figure 1 to determine σ_T_ at 532 nm for each Z-scan.^99^ Extinction coefficients obtained via UV–visible absorption measurements were used to calculate σ_g_ according to eq 4.

4 $$\sigma \left({cm}^{2}\right)=\frac{2.3\times 10^{8}\left(L^{-1}cm^{-3}\right)}{6.02\times 10^{23}\left(mol^{-1}\right)}\varepsilon \left(L\;mol^{-1}cm^{-1}\right)$$

**Figure 10:**
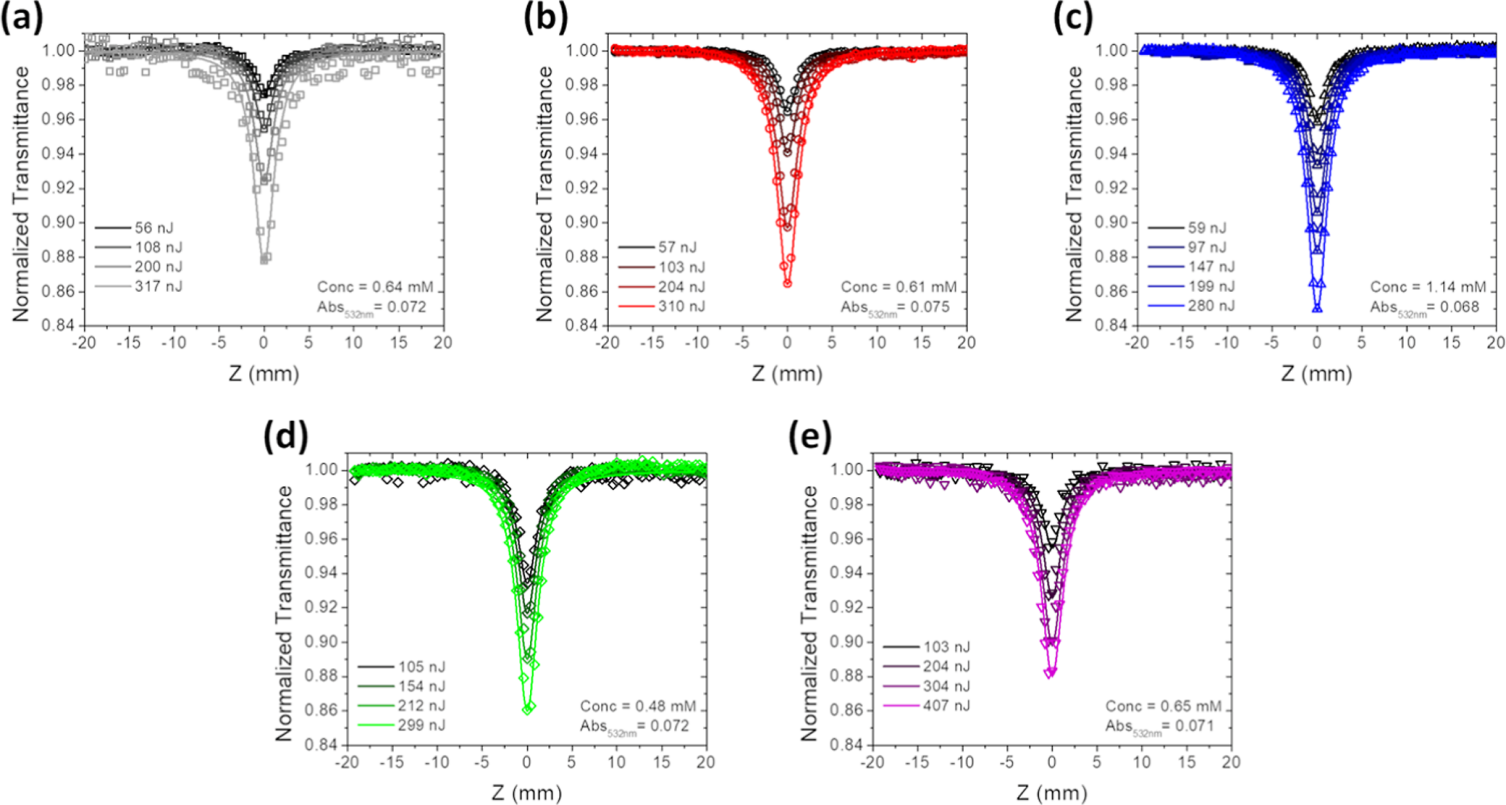
Open-aperture ns Z-scans of (a) **1**; (b) **2**; (c) **3**; (d) **4**; and (e) **5** in toluene. (λ_ex_ = 532 nm, fwhm = 7.2 ns).

**Figure 11:**
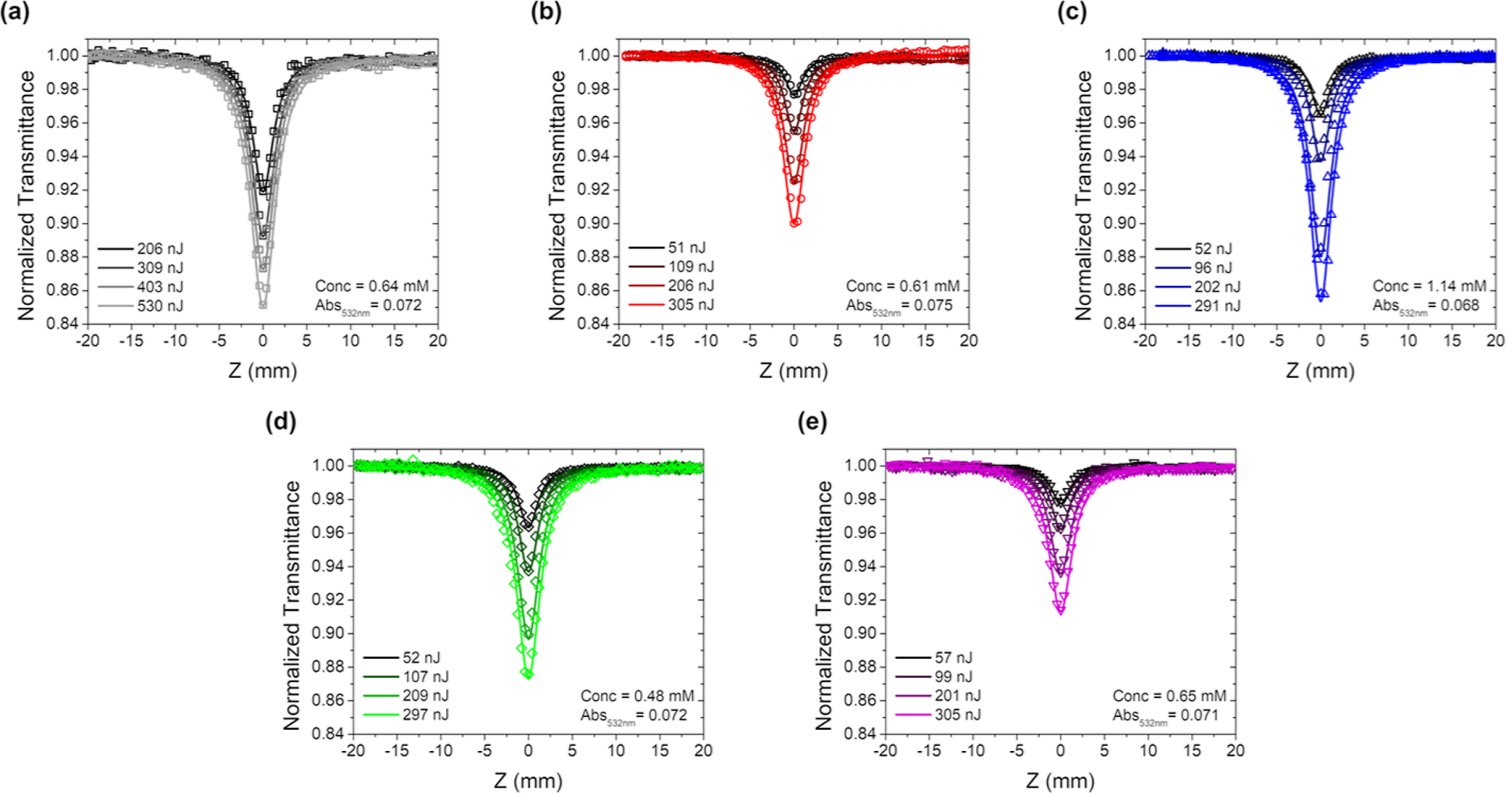
Open-aperture ps Z-scans of (a) **1**; (b) **2**; (c) **3**; (d) **4**; and (e) **5** in toluene. (λ_ex_ = 532 nm, fwhm = 18 ps).

The average σ_T_ is reported in Table 5, and a comparison of the ground state and ESA cross sections is depicted in Figure 12.

**Table 5:** Ground State and Excited State Cross Sections at 532 nm for Complexes **1**–**5** Obtained From Z-Scan Analysis. (λ_ex_ = 532 nm)

complex	σ_g_/10^–18^ cm^2^	σ_T_/10^–18^ cm^2^ ^a^	σ_T_/10^–18^ cm^2^ ^b^	σ_T_/σ_g_
**1**	4.21	21(1)	22.9(3)	5.3
**2**	4.94	24.5(3)	23(4)	4.9
**3**	3.10	22.4(3)	21.5(9)	7.2
**4**	5.48	29.1(9)	30.1(3)	5.5
**5**	6.11	17.2(4)	16.4(3)	2.8

a From ns Z-scans (fwhm = 7.2 ns).

b From ps Z-scans (fwhm = 18 ps).

**Figure 12:**
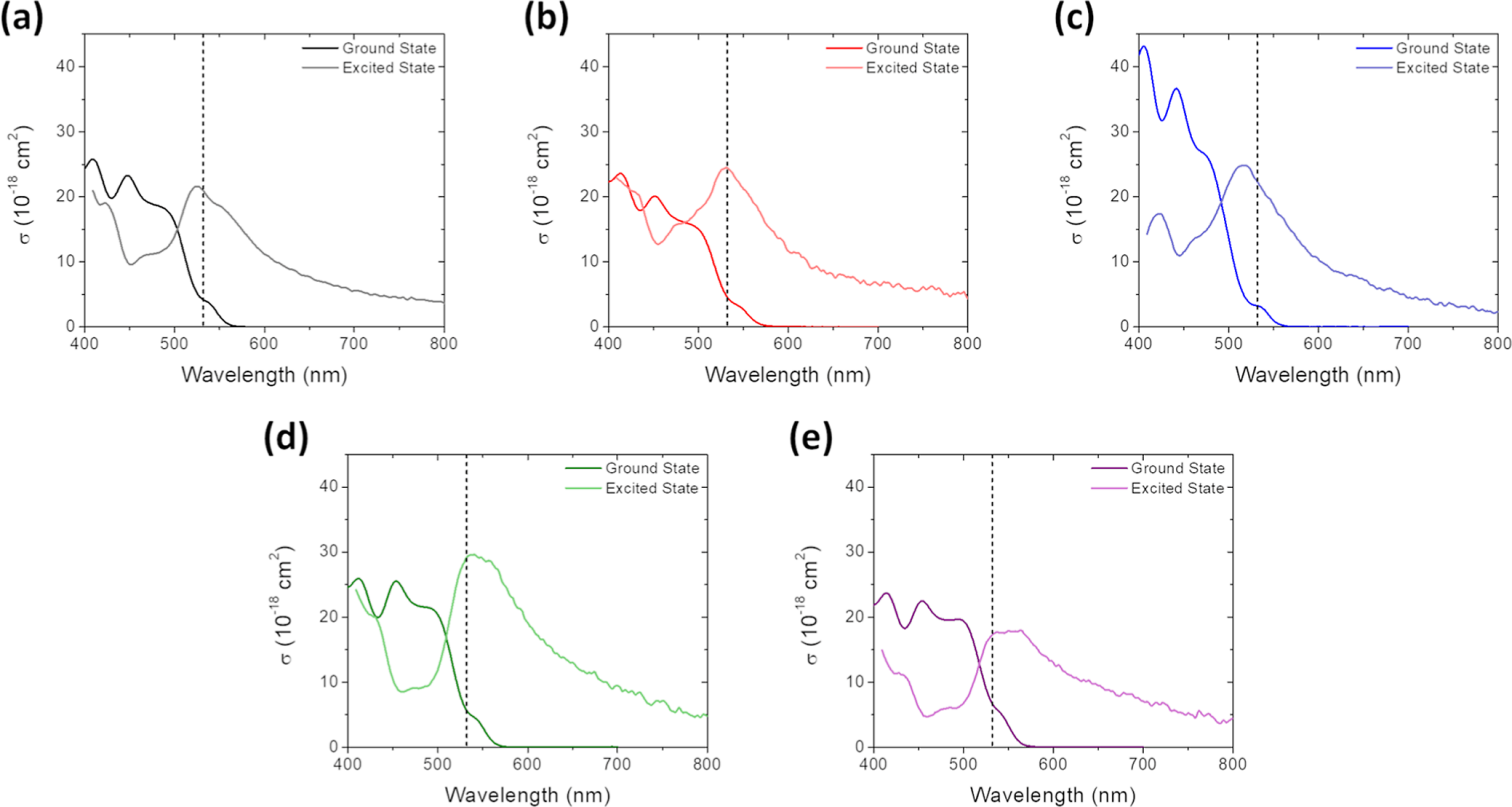
Ground state (dark line) and excited state (light line) absorption cross sections for (a) **1**; (b) **2**; (c) **3**; (d) **4**; and (e) **5**. ESA was obtained from the 5 ns time slice of the TA scans and the cross section for each wavelength was calculated based on σ_T_ at 532 nm. The dashed vertical line intersects the plots at 532 nm.

The ratio of σ_T_/σ_g_ is a common figure of merit used to determine the effectiveness of RSA.^60^ While there is no direct correlation among the magnitudes of σ_T_, σ_g_, and *E*_00_, σ_T_/σ_g_ tends to decrease as the sample absorbance red-shifts for this series of compounds, with **3** exhibiting the strongest RSA capabilities and **5** the weakest. These Ir­(III) complexes undergo rapid intersystem crossing to the triplet as evidenced by both nanosecond and picosecond Z-scan measurements and subsequent fitting (Figure 11, Table 5). The triplet quantum yield was set to unity for the purpose of σ_T_ calculations due to the ultrafast intersystem crossing rates and near-unity triplet yields of Ir­(III) chromophores.^34–40^

The σ_T_/σ_g_ values of complexes **1**–**5** were compared to recent works of related Ir­(III) complexes (Table 6).^60,105–107^ Though several other Ir­(III) complexes have exhibited RSA capabilities at 532 nm^108–111^ in recent years, σ_T_ was not calculated in the papers, making direct comparison between those complexes and the Br-pbt complexes reported herein unfeasible. The σ_T_/σ_g_ values for the related iridium complexes range from 8.34 to 88, depending on the complex. It is worth noting, however, that the complex reported by Shensky et al.^60^ with σ_T_/σ_g_ of 88 has an extremely weak ground state absorbance at 532 nm (σ_g_ = 0.26 × 10^–18^ cm^2^), contributing to its high apparent RSA effectiveness. Compared to other reported Ir­(III) chromophores, complexes **1**–**5** are less effective RSA agents but present a methodical way to tune HOMO–LUMO gaps through rational ligand design in future studies.

**Table 6:** Ground State and Excited State Cross Sections at 532 nm for Related Iridium­(III) Complexes (λ_ex_ = 532 nm).

complex	σ_g_/10^–18^ cm^2^	σ_T_/10^–18^ cm^2^	σ_T_/σ_g_
(quqo)Ir(C^N^1^)_2_PF_6_ ^107^	4.14	88.5	21.4
(quqo)Ir(C^N^2^)_2_PF_6_ ^107^	3.43	28.6	8.34
(quqo)Ir(C^N^3^)_2_PF_6_ ^107^	3.69	44	11.9
(quqo)Ir(C^N^4^)_2_PF_6_ ^107^	3.77	33.5	8.89
Ir-1 ^106^	8.2	168	20.5
Ir-2 ^106^	8.1	183	22.6
Ir3 ^105^	26.74	78.27	2.93
Ir4 ^105^	15.28	33.78	2.21
Ir5 ^105^	38.20	110.78	2.90
TCQ[Ir^3+^(ppz)_2_][Bibr ref60]	0.26	23	88

## 4 Discussion

Electrochemical, theoretical, and photophysical properties of complexes **1**–**5** clearly show that the position of bromo substitution matters. Additionally, XRD data suggest that the variances in the photophysical and electrochemical properties are more likely due to electronic effects of the bromo location than from structural differences between the complexes, as the structures for **1**–**5** are statistically similar. The HOMO–LUMO gaps calculated from electrochemical, theoretical, and photophysical measurements all loosely trend with each other (Figures S23), supporting the validity of the DFT calculations as they relate to experimental data.

As noted in Section 3.2, Ir^IV/III^ reduction exhibited more variation than ligand reduction, which was relatively consistent for the brominated chromophores, suggesting that the HOMO–LUMO gap is largely dictated by HOMO mixing with the bromine. This same trend was observed via DFT calculations: the HOMO levels of the brominated complexes varied while the LUMO levels were approximately the same. Closer inspection of the orbital density maps of complexes **1**–**5** reveals that on the ligand the HOMO is localized on the iridium and the cyclometalating ring but does not extend to the benzothiazole moiety. Additionally, complex **2**, where the bromine is positioned *para* to the Ir­(III) center, is the only case in which significant density is localized on the bromine itself. These variations in bromine coupling and proximity to the HOMO likely account for the wide variation in electrochemically and theoretically calculated HOMO energies. Meanwhile, the orbital density extends across the entire pbt ligand in the LUMO but does not couple to the bromine for any complex. This is also consistent with the observation that electrochemically and theoretically calculated LUMO energies are largely the same for the brominated chromophores.

While the photophysics do not reveal where the HOMO/LUMO levels lie energetically, they can be used to calculate the gap between HOMO and LUMO. *E*_00_ values obtained at 77 K are analogous to the HOMO–LUMO gap and do closely trend with DFT calculations (Figure S23), following the trend **3** > **1** > **2** > **4** = **5** which matches what is seen with the absorbance shifts. Additionally, the HOMO–1 to LUMO gaps (Table 3) are within 0.01 eV of the HOMO–LUMO gap, making the appearance of two low-energy TD-DFT transitions unsurprising (Tables S6–S7).

It is worth noting that unlike the other complexes, **2** does not follow many of the expected photophysical trends in the series, which may be explained by MO analysis and further evaluation of the FCLSA parameters. Though there is generally very little variation in the orbital density maps of complexes **1**–**5**, closer inspection of the 20 MOs depicted for **2**–**5** (Figure 7) reveals only a single case where MO density is located on the bromine substituent; there is significant density localized on bromine in the HOMO of **2**, possibly due to its position with regard to the metal center. The noted deviation provides a mechanism for direct electronic communication between the metal and bromine only for **2**, which fundamentally alters its photophysical properties relative to complexes **3**–**5** as this is not a type of electronic communication available to them. Consequently, bromine-donated electron density into the metal–ligand system center may fundamentally alter photophysical properties, perhaps by impacting the reorganizational energy of the transition pair, leading to higher rates of nonradiative decay as compared to the other chromophores that do not demonstrate this resonance donation.

Franck–Condon Line Shape Analysis further supports that the reorganizational energy may be impacted by the location of bromine on the ligand. Previous works have shown that the Huang–Rhys factor, *S*, corresponds to vibrational coupling and is proportional to the square of the distortion of molecular coordinates.^112^ As a result, larger *S* values correspond to more distortion in the excited state and can lead to deviations from the energy gap law^112–114^ which assumes little to no excited state distortion.^114,115^ In general (and again with the exception of **2**), the Ir­(III) chromophores reported here trend opposite of the energy gap law, with smaller *E*_00_ values instead corresponding with higher quantum yields and lower nonradiative decay rate constants. The Huang–Rhys values for the complexes (Tables S1 and S2) may be able to rationalize this deviation; compounds with higher *S* values also tend to have higher rates of nonradiative decay. Based on these observations, it seems likely that bromine-donated electron density into the metal–ligand system may indeed impact the reorganizational energy of the transition for complex **2**.

## 5 Conclusions

3-, 4-, 6-, and 7-bromo-substituted RSA chromophores were synthesized, and their electronic and photophysical properties were characterized in this work.

X-ray diffraction studies indicated that the bromo-substituted iridium complexes exhibit a distorted octahedral geometry with structure metrics nearly identical to those of the parent pbt complex. The lack of variance in atom bond lengths directly coordinated to iridium suggests that the variances in the photophysical properties are more likely due to electronic effects of the bromo location in different positions rather than from structural differences between the complexes.

TA spectroscopy indicated that these complexes demonstrated enhanced absorption in the excited state and indicated that they were good candidates as RSA chromophores, which was further verified by open aperture Z-scans. RSA effectiveness in the brominated complexes was quantified and compared to literature studies using the ratios of excited state to ground state absorption cross sections at 532 nm. Overall, **3** demonstrated the highest capacity for RSA, followed by **4**, **1**, **2**, and **5**.

Investigations are currently under way to evaluate why these complexes follow the described RSA trend and why **2** does not follow the photophysical trends anticipated from the photophysically calculated energy gaps between the lowest vibrational levels of the ground- and excited-states. Electrochemical analysis and computational modeling provide some insight in the latter case, as both indicate that addition of bromine does not significantly affect the energy of the LUMOs in the complexes, though they do cause variations in the HOMOs as observed. These effects are seen most prominently in **2**, which has more HOMO electron density localized on the bromine than **3**–**5**, and in **3**, which is the most blue-shifted of the chromophores.

Overall, these results show that the position of substituents on a ligand matters when designing RSA-capable chromophores. Using a stronger electron-donating or withdrawing substituent may prove to further exaggerate the positional effects of substitution. Furthermore, C­(sp^2^)-Br functionalized compounds are versatile palladium cross-coupling partners. Their inclusion at various locations of these iridium RSA complexes may likewise serve as a foundation for other possible applied and designer material applications when coupling partners are rationally selected.

## Supplementary Material













## Notes

Additional experimental procedures, ^1^H NMR spectra of 2–5, CV, ESI-MS data, extinction coefficients, FCLSA data, emission decay traces, TA decay traces and lifetime data, open-aperture Z-scan data, and HOMO–LUMO gaps plotted vs various metrics (PDF)
Computational optimized geometry coordinates for 3-Brpbt (XYZ)
Computational optimized geometry coordinates for 4-Brpbt (XYZ)
Computational optimized geometry coordinates for 6-Brpbt (XYZ)
Computational optimized geometry coordinates for 7-Brpbt (XYZ)
Computational optimized geometry coordinates for pbt (XYZ)

## Abbreviations

TMC: transition metal complexes
NLO: nonlinear optics
RSA: reverse saturable absorption
pbt: phenylbenzothiazole
acac: acetyl acetonate
**1**: 2-phenylbenzo­[^*d*^]­thiazole
**2**: 2-(3-bromophenyl)­benzo­[^*d*^]­thiazole
**3**: 2-(4-bromophenyl)­benzo­[^*d*^]­thiazole
**4**: 2-(6-bromophenyl)­benzo­[^*d*^]­thiazole
**5**: 2-(7-bromophenyl)­benzo­[^*d*^]­thiazole
RT: room temperature
SiNc: silicon naphthalocyanine
TBAOH: tetra-*N*-butylammonium hydroxide
TBAPF_6_: tetra-*N*-butylammonium hexafluorophosphate
DCM: dichloromethane
THF: tetrahydrofuran
CDCl_3_: deuterated chloroform
DMSO: dimethyl sulfoxide
NMR: nuclear magnetic resonance
MS: mass spectrometry
ESI: electrospray ionization
TOF: time-of-flight
IR: infrared
XRD: X-ray diffraction
TCSPC: time correlated single photon counting
TA: transient absorption
DFT: density functional theory
TD-DFT: time dependent DFT
MO: molecular orbital
HOMO: highest occupied molecular orbital
LUMO: lowest unoccupied molecular orbital
CV: cyclic voltammogram/voltammetry
GC: glassy carbon
$E_{1/2}^{{}^{\circ}\prime }$: formal reduction potential
$E_{p,c}^{{}^{\circ}\prime }$: cathodic formal peak potential
$E_{p,a}^{{}^{\circ}\prime }$: anodic formal peak potential
Δ $E_{p}^{{}^{\circ}\prime }$: peak-to-peak separation
*L*_*π*_^0/–1^: ligand-based reduction
Ir^IV/III^: Iridium-based reduction
Δ*E*_HOMO–LUMO_: electrochemically measured HOMO–LUMO gap
FCLSA: Franck–Condon Line Shape Analysis
S (FCLSA): Huang–Rhys factor
I­(ν̅): intensity as a function of wavenumber
ν̅: wavenumber
N: number of quantum levels
ν: vibronic quantum number
*E*_0_: 0–0 energy gap
*E*_00_: 0–0 energy gap at 77 K
Δν_1/2_: full width at half-maximum
ℏω: quantum spacing parameter
M: medium frequency vibrational mode
L: low-frequency vibrational mode
ϕ: quantum yield
ϕ_*f*_: quantum yield of fluorescence
ϕ_em_: quantum yield of emission
ϕ_T_: triplet quantum yield
*S* (quantum yield): sample
R: reference
I (quantum yield): integrated emission intensity
A: absorbance at 500 nm
η: refractive index of the solvent
O.D.: optical density
MLCT: metal to ligand charge transfer
em: emission
ex: excitation
S_0_: ground state singlet
ε: extinction coefficient
S_1_: first excited singlet state
S_n_: higher lying excited singlet states
T_1_: lowest lying excited triplet state
T_*n*_: higher lying excited triplet states
*k*_ISC_: rate constant for intersystem crossing
*k*_nr_: rate constant for nonradiative decay
*k*_r_: rate constant for radiative decay
ESA: excited state absorption
fwhm: full width at half-maximum
σ_g_: ground-state absorption cross section
σ_S_: excited singlet-state absorption cross section
σ_T_: excited triplet-state absorption cross section
σ_T_/σ_g_: RSA capacity/effectiveness.
