# Supplementary material for: Bromo-Substituted Phenylbenzothiazole Cyclometalating Ligands for the Development of Reverse Saturable Absorption Materials
Source: Inorg Chem. 2025 Jul 11;64(29):14952–67. doi: 10.1021/acs.inorgchem.5c01726 (PMC12308814; doi:10.1021/acs.inorgchem.5c01726)
Supplement: Supplementary file 1 [file ic5c01726_si_001.pdf]

Supplemental Information

Bromo-Substituted Phenylbenzothiazole Cyclometalating Ligands for the Development of Reverse Saturable Absorption Materials

Erica S. Knorr,[a] Jordan C. Kelly,[a] Daniel P. Harrison,[b] Catherine J. Fabiano,[c] Caitlin G. Bresnahan,[d] Ryan B. Gaynor,[a,e] Jack M. Harrison,[e] Nanki Verma,[e] Trenton R. Ensley,[a] Ryan M. O'Donnell,[a] Kenneth J. Smith,[f] Peter Y. Zavalij,[g] Thomas N. Rohrabaugh, Jr.,[a*] Chi K. Nguyen,[e*] Victor A. Jaffett[e*]

[a] - U.S. Army Combat Capabilities Development Command Army Research Laboratory, 2800 Powder Mill Rd, Adelphi, MD 20783, United States
[b] - Virginia Military Institute, Department of Chemistry, 401 Maury-Brooke Hall, Lexington, VA 24450, United States
[c] - Rice University, Department of Chemistry, 6100 Main St, Houston, TX 77251, United States
[d] - U.S. Army Corps of Engineers, Engineer Research and Development Center, 3909 Halls Ferry Rd, Vicksburg, MS 39180, United States
[e] - United States Military Academy, Department of Chemistry and Life Science, 753 Cullum Rd, West Point, NY 10996, United States
[f] - Fibertek, Inc., 13605 Dulles Technology Dr, Herndon, VA 20171, United States
[g] - University of Maryland, Department of Chemistry and Biochemistry, 8051 Regents Drive, College Park, MD 20742, United States
[*] - corresponding authors

* Thomas N. Rohrabaugh, Jr., email address: thomas.n.rohrabaugh.civ@army.mil

* Chi K. Nguyen, email address: chi.nguyen@westpoint.edu

* Victor A. Jaffett, email addres: victor.jaffett@westpoint.edu

Table of Contents

1. Synthesis of 3-Brpbt and $[Ir(L)_2(m\text{-}Cl)]_2$ complexes……………………..……… S2
2. Physical Characterization………………………………………………………… S3
3. Cyclic Voltammetry.……………………………………………….………... S6
4. Extinction Coefficients……………………………………………………….. S8
5. Franck Condon Line Shape Analysis……………………………………………….. S11
6. Miscellaneous Photophysical Data…...………………………………………..…. S14
7. Computational Details………………………………………………………. S17

1. Synthesis of 3-Brpbt and [Ir(L)$_2$(m-Cl)]$_2$ complexes

3-Brpbt (2-(3-bromophenyl)-benzo[^{d}]thiazole): 1.606 g of 3-bromobenzoic acid (7.99 mmol) and 3 g of tetrabutylamonium bromide were combined in a microwave vial followed by 2.2 mL of triphenylphosiphite (8.4 mmol) and 1.5 mL of 2-aminothiophenol (14 mmol). The vial was capped and loaded into the microwave reactor. The system was subjected to microwave irradiation heating to 160 °C for 20 minutes, then held at 160 °C for 15 minutes more. After completion, the system was cooled to room temperature and reaction mixture was suspended in 200 mL of saturated aqueous sodium carbonate and stirred for 30 minutes. The resulting precipitate was collected by vacuum filtration and filter cake rinsed with water (3 x 30 mL). The filter cake was dissolved in 200 mL of dichloromethane and filtered to remove insoluble impurities. The filtrate was collected, and solvent removed by rotary evaporation resulting 1.999 g (6.86 mmol, 85.9%) of white solid and used without further purification. ^{1}H NMR (400 MHz, $CDCl_3$) δ 8.28 (t, J = 1.8 Hz, 1H), 8.09 (d, J = 8.2 Hz, 1H), 8.00 (dt, J = 7.8, 1.3 Hz, 1H), 7.92 (d, J = 8.0 Hz, 1H), 7.62 (ddd, J = 8.0, 1.9, 0.9 Hz, 1H), 7.54 – 7.49 (m, 1H), 7.44 – 7.39 (m, 1H), 7.37 (t, J = 7.9 Hz, 1H).

[Ir(3-Brpbt)$_2$(m-Cl)]$_2$: 580 mg of 2-(3-bromophenyl)-benzo[^{d}]thiazole (2.00 mmol) and 352 mg of iridium(III) trichloride trihydrate (1.00 mmol) were combined in a microwave reactor vial followed by 10 mL of 2-methoxyethanol/water (3:1) and capped. The mixture was loaded into the microwave reactor and heated to 150 °C for 1.5 hours. After completion, the reaction mixture was diluted with 3 mL of 3M HCl(aq) and stirred for 15 minutes. The precipitate was collected by vacuum filtration and washed with water, ethanol, diethyl ether, and hexanes. The filter cake was dried in a vacuum oven overnight, resulting in 532 mg (0.330 mmol, 66.0%) of orange solid and used without further purification.

[Ir(4-Brpbt)$_2$(m-Cl)]$_2$: 581 mg of 2-(4-bromophenyl)-benzo[^{d}]thiazole (2.00 mmol) and 353 mg of iridium(III) trichloride trihydrate (1.00 mmol) were combined in a microwave reactor vial followed by 10 mL of 2-methoxyethanol/water (3:1) and capped. The mixture was loaded into the microwave reactor and heated to 150 °C for 1.5 hours. After completion, the reaction mixture was diluted with 3 mL of 3M HCl(aq) and stirred for 15 minutes. The precipitate was collected by vacuum filtration and washed with water, ethanol, diethyl ether, and hexanes. The filter cake was dried in a vacuum oven overnight, resulting in 695 mg (0.431 mmol, 86.2%) of orange solid and used without further purification.

[Ir(6-Brpbt)$_2$(m-Cl)]$_2$: 581 mg of 2-(4-bromophenyl)-benzo[^{d}]thiazole (2.00 mmol) and 353 mg of iridium(III) trichloride trihydrate (1.00 mmol) were combined in a microwave reactor vial followed by 10 mL of 2-methoxyethanol/water (3:1) and capped. The mixture was loaded into the microwave reactor and heated to 150 °C for 1.5 hours. After completion, the reaction mixture was diluted with 3 mL of 3M HCl(aq) and stirred for 15 minutes. The precipitate was collected by vacuum filtration and washed with water, ethanol, diethyl ether, and hexanes. The filter cake was dried in a vacuum oven overnight, resulting in 695 mg (0.431 mmol, 86.2%) of orange solid and used without further purification.

[Ir(7-Brpbt)$_2$(m-Cl)]$_2$: 580 mg of 2-(4-bromophenyl)-benzo[^{d}]thiazole (2.00 mmol) and 353 mg of iridium(III) trichloride trihydrate (1.00 mmol) were combined in a microwave reactor vial followed by 10 mL of 2-methoxyethanol/water (3:1) and capped. The mixture was loaded into the microwave reactor and heated to 150 °C for 1.5 hours. After completion, the reaction mixture was diluted with 3 mL of 3M HCl(aq) and stirred for 15 minutes. The precipitate was collected by vacuum filtration and washed with water, ethanol, diethyl ether, and hexanes. The filter cake was dried in a vacuum oven overnight, resulting in 714 mg (0.443 mmol, 88.6%) of orange solid and used without further purification.

2. Physical Characterization

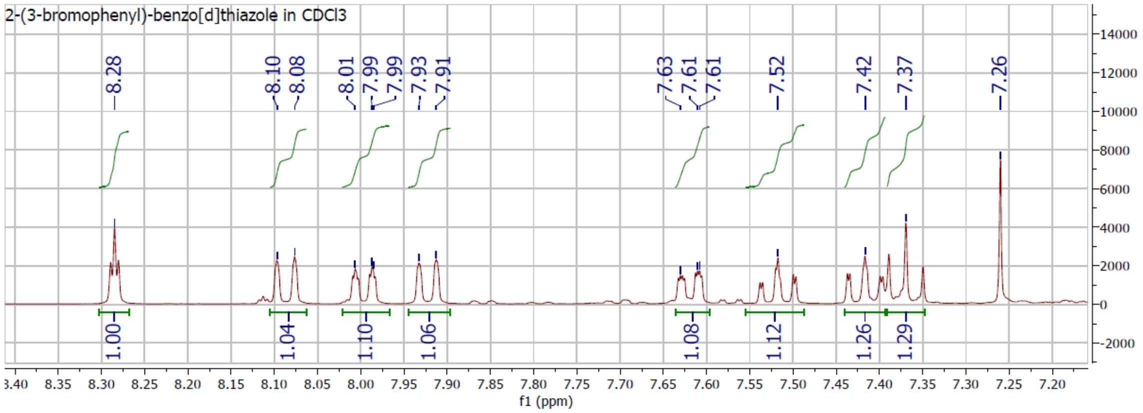

Figure S1. ¹H NMR of **3-Brpbt** in $CDCl_3$.

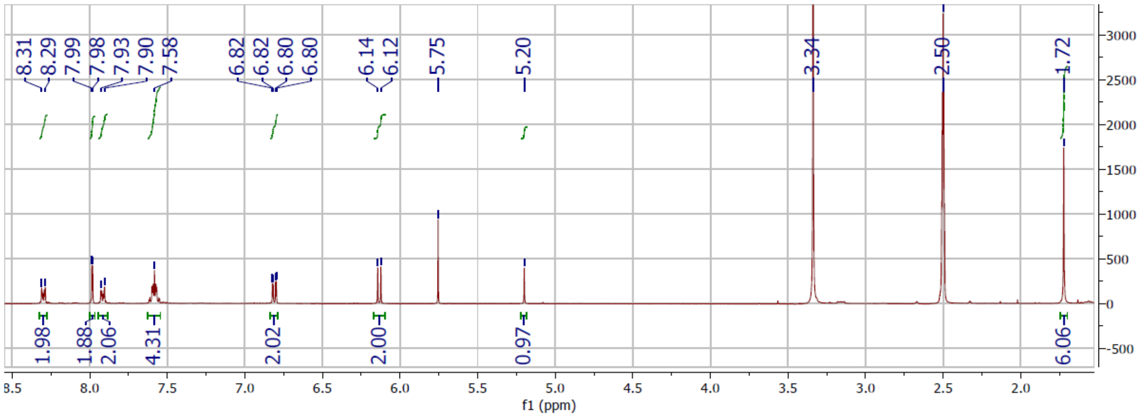

Figure S2. NMR of **2** in DMSO-d_6.

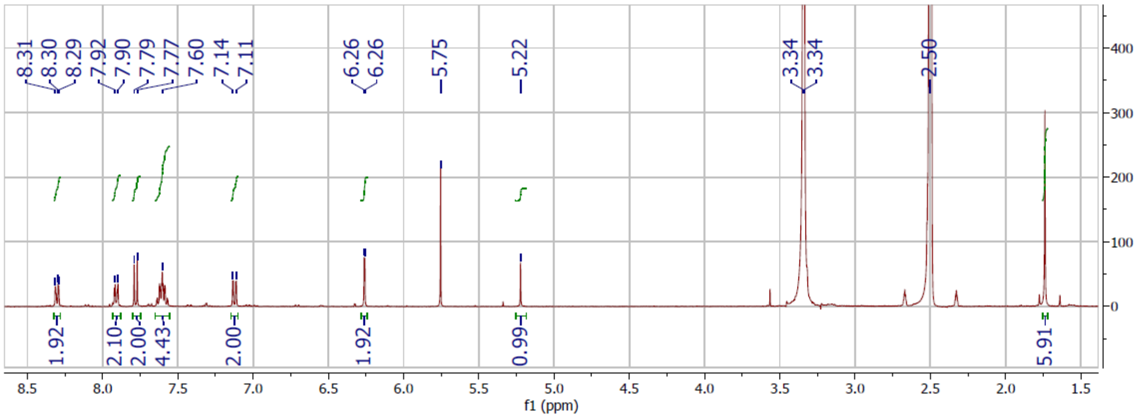

Figure S3. NMR of **3** in DMSO-d_6.

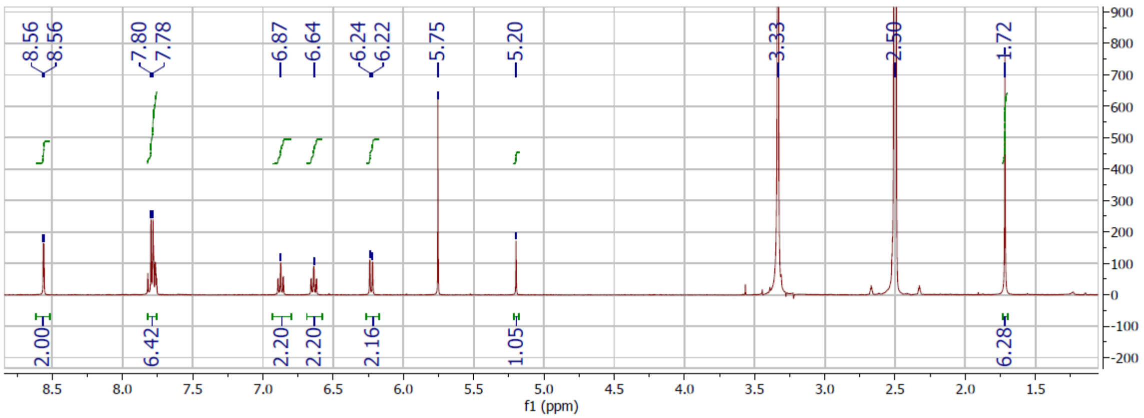

Figure S4. NMR of **4** in DMSO-d_6.

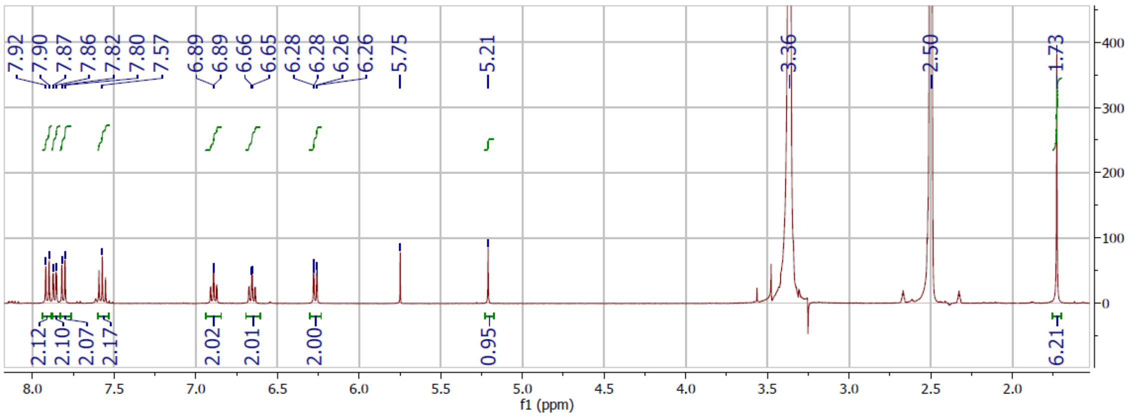

Figure S5. NMR of **5** in DMSO-d_6.

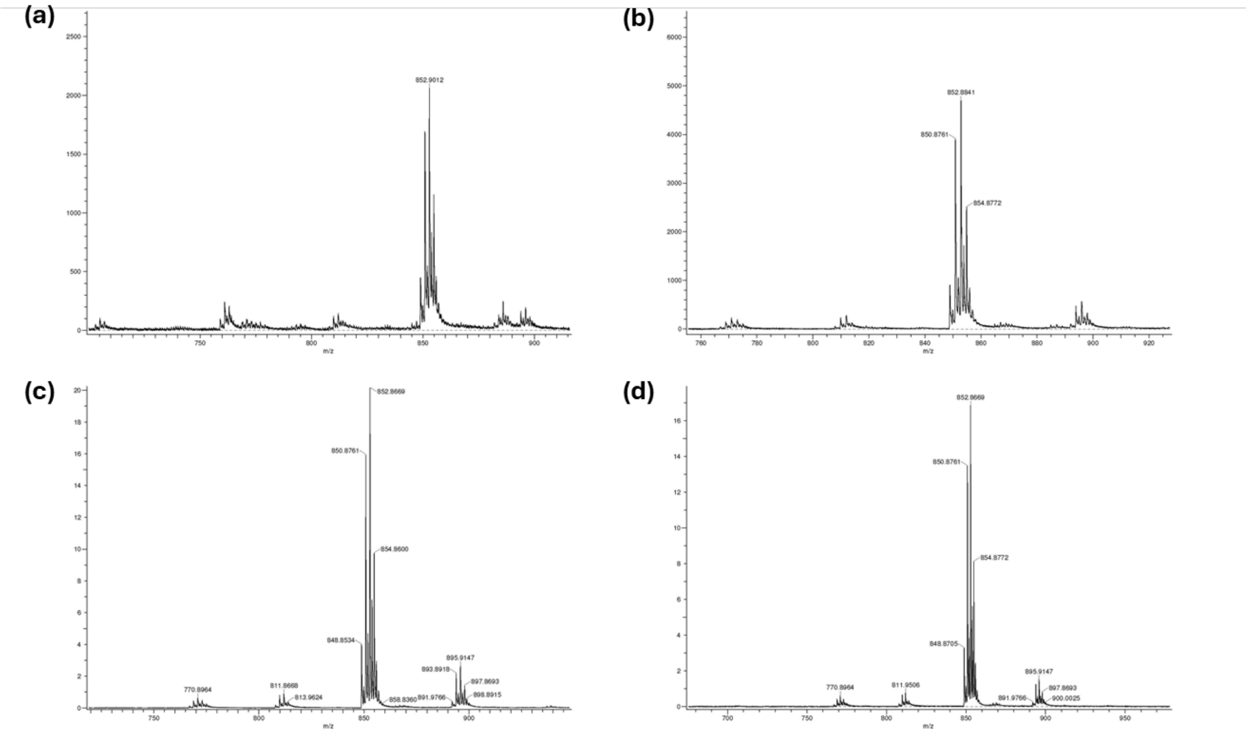

Figure S6. ESI Mass spec of chromophores **2** (a), **3** (b), **4** (c)**,** and **5** (d).

3. Cyclic voltammetry

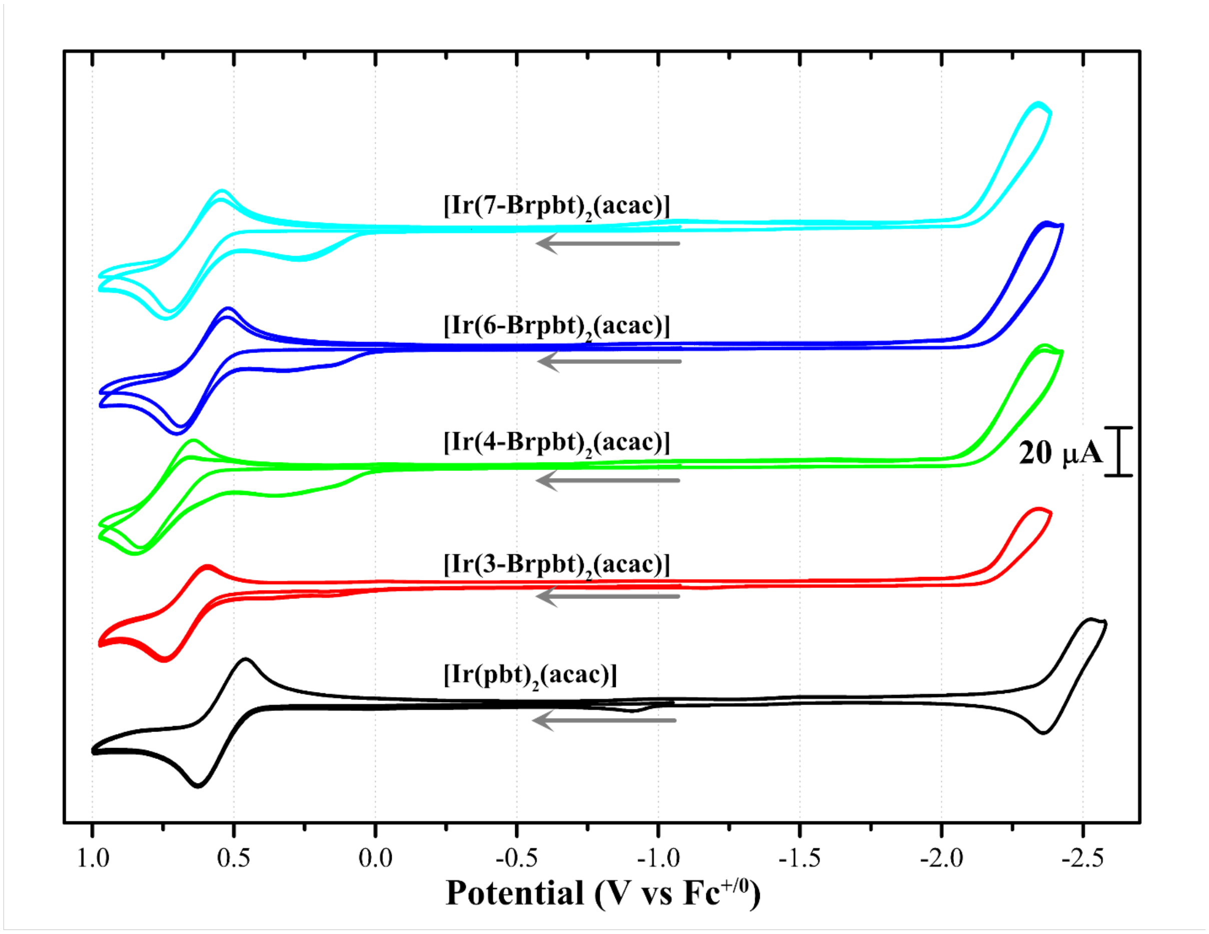

Figure S7. Cyclic voltammograms of the title complexes collected at 100 mV/s scan rate, in anhydrous THF containing 0.1 M $TBAPF_6$ using IR compensation. The three cyclic voltammograms cycle were collected by scanning anodically from an initial potential of -1.1 V (vs $Fc^{+/0}$) to a switching potential of about +1.0 V, which was followed by a cathodic scan to a switching potential 0.05-0.1 V beyond the $E_{p,c}$°' (E°' of **1**) of the complexes and back to -1.1V. Gray arrows indicate initial scan direction. Traces: **1** (1.9 mM, black); **2** (1.9 mM, red); **3** (1.8 mM green); **4** (1.8 mM, dark blue); **5** (2.0 mM, cyan).

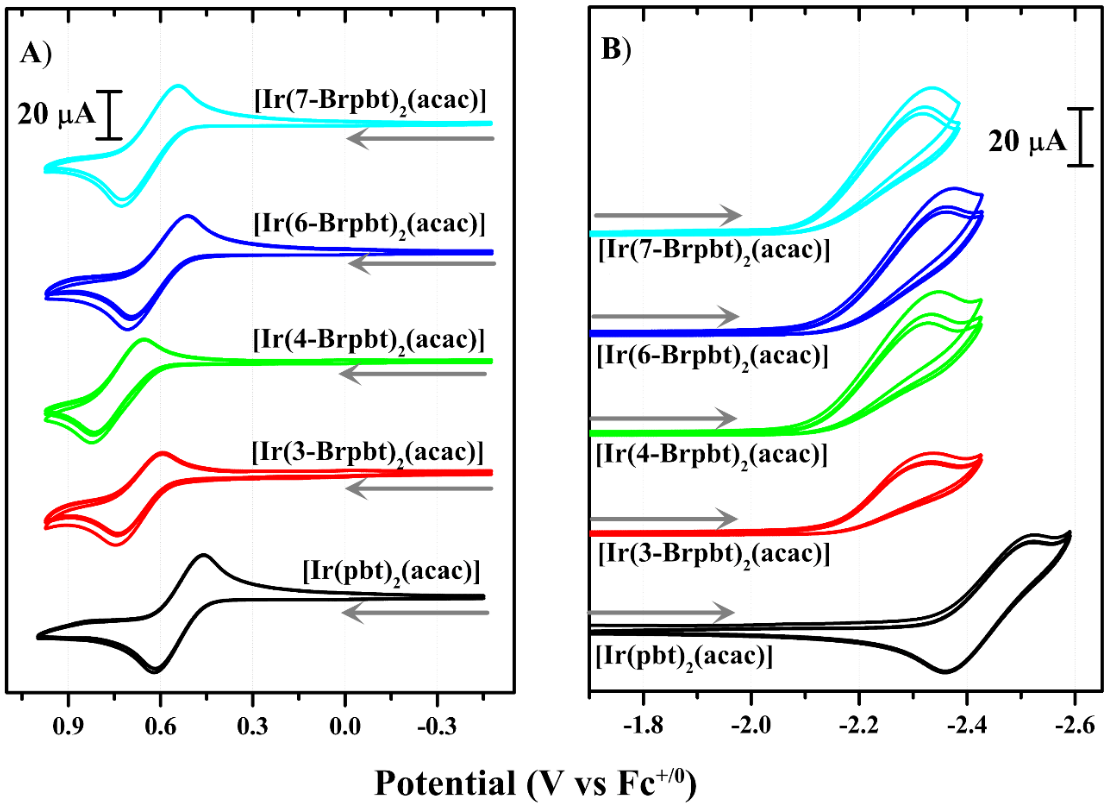

Figure S8. Cyclic voltammograms of the title complexes collected at 100 mV/s scan rate, in anhydrous THF containing 0.1 M $TBAPF_6$ using IR compensation. Panel A: Cyclic voltammograms of the title complexes collected by scanning anodically for three cyclic voltammogram cycles with an initial potential of approximately -0.4 V (vs $Fc^{+/0}$) to a switching potential of about +1.0 V and returning to about -0.4 V. Panel B: CVs of the title complexes collected by scanning anodically from about -1.1 V to 0.05-0.1 V beyond the $E_{p,c}°'$ (E°' of **1**). Gray arrows indicate initial scan direction. Traces: **1** (1.9 mM, black); **2** (1.9 mM, red); **3** (1.8 mM green); **4** (1.8 mM, dark blue); **5** (2.0 mM, cyan).

4. Extinction Coefficients

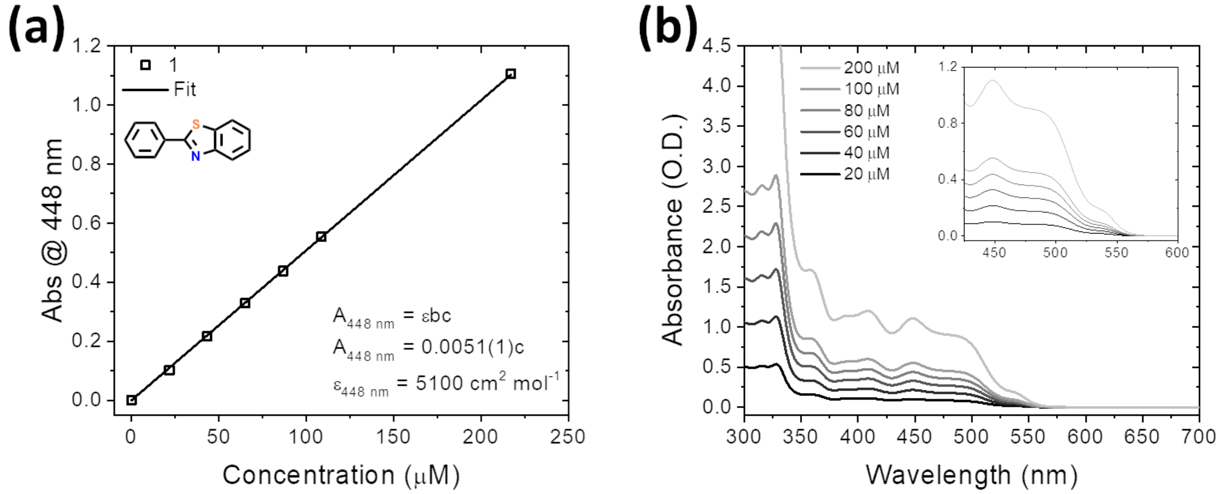

Figure S9. (a) Extinction coefficient Beer-Lambert plot at the highest energy MLCT band (λ = 448 nm) for **1** in toluene; (b) Absorption spectra of **1** in toluene at various concentrations.

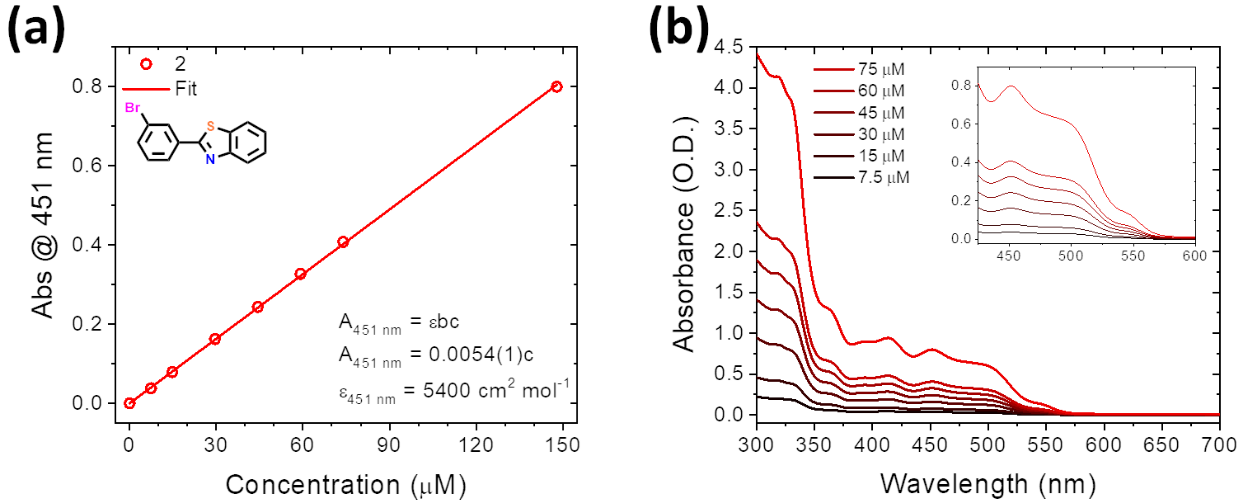

Figure S10. (a) Extinction coefficient Beer-Lambert plot at the highest energy MLCT band (λ = 451 nm) for **2** in toluene; (b) Absorption spectra of **2** in toluene at various concentrations.

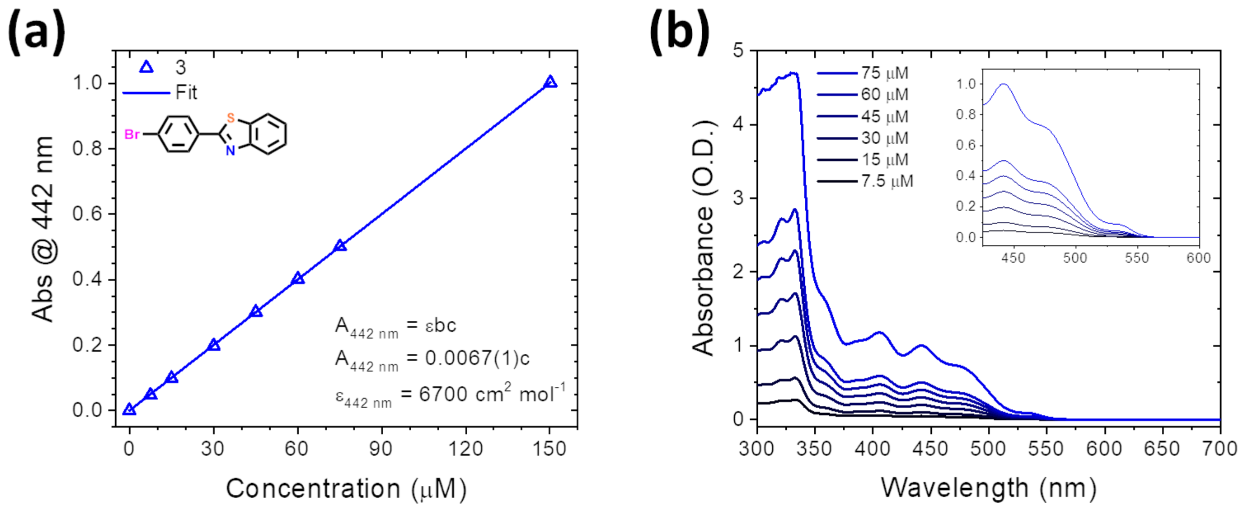

Figure S11. (a) Extinction coefficient Beer-Lambert plot at the highest energy MLCT band (λ = 442 nm) for **3** in toluene; (b) Absorption spectra of **3** in toluene at various concentrations.

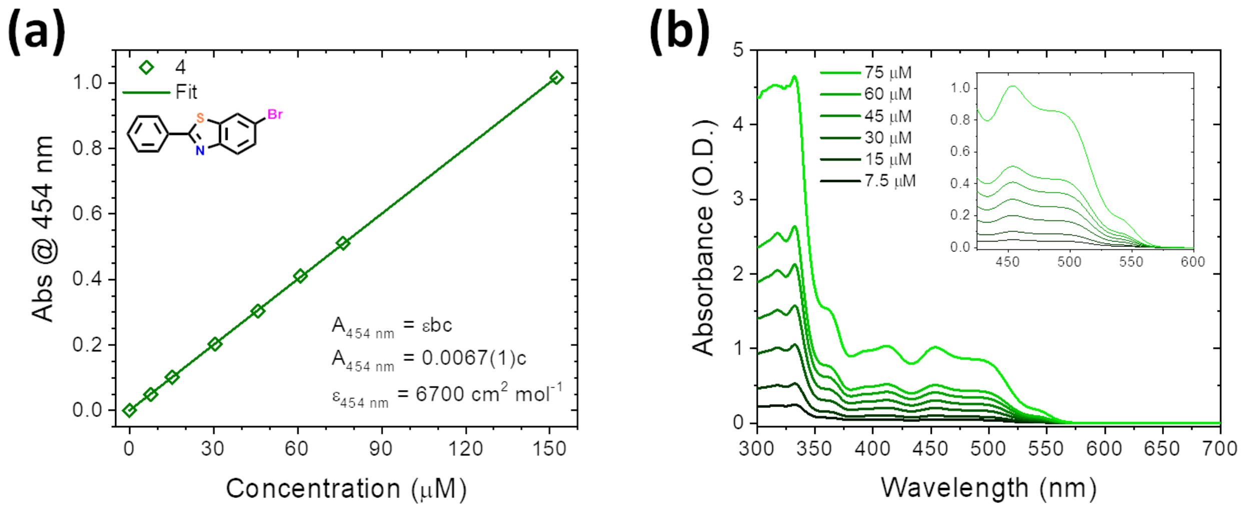

Figure S12. (a) Extinction coefficient Beer-Lambert plot at the highest energy MLCT band (λ = 454 nm) for **4** in toluene; (b) Absorption spectra of **4** in toluene at various concentrations.

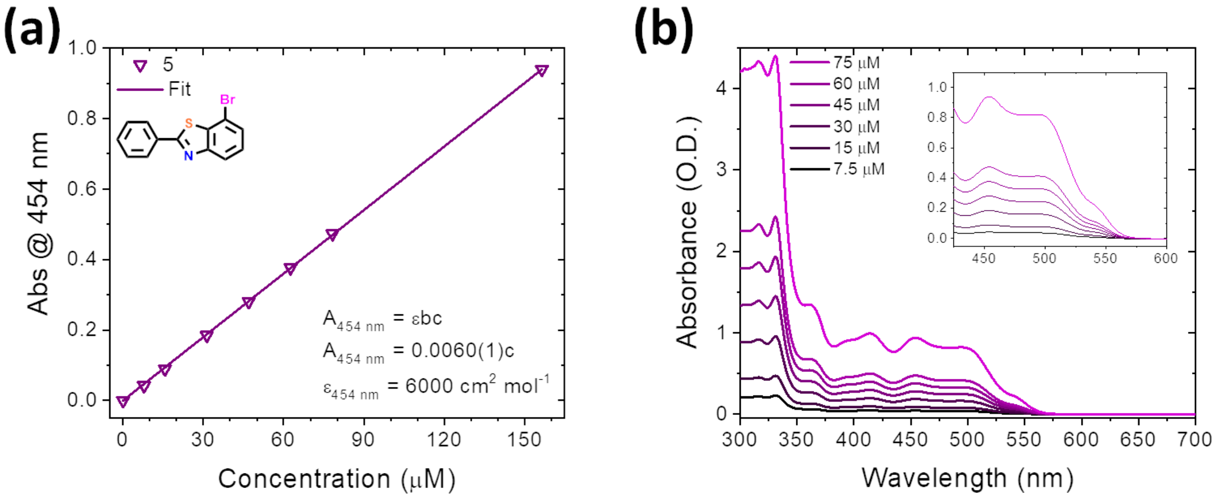

Figure S13. (a) Extinction coefficient Beer-Lambert plot at the highest energy MLCT band (λ = 454 nm) for **5** in toluene; (b) Absorption spectra of **5** in toluene at various concentrations.

5. Franck-Condon Line Shape Analysis (FCLSA)

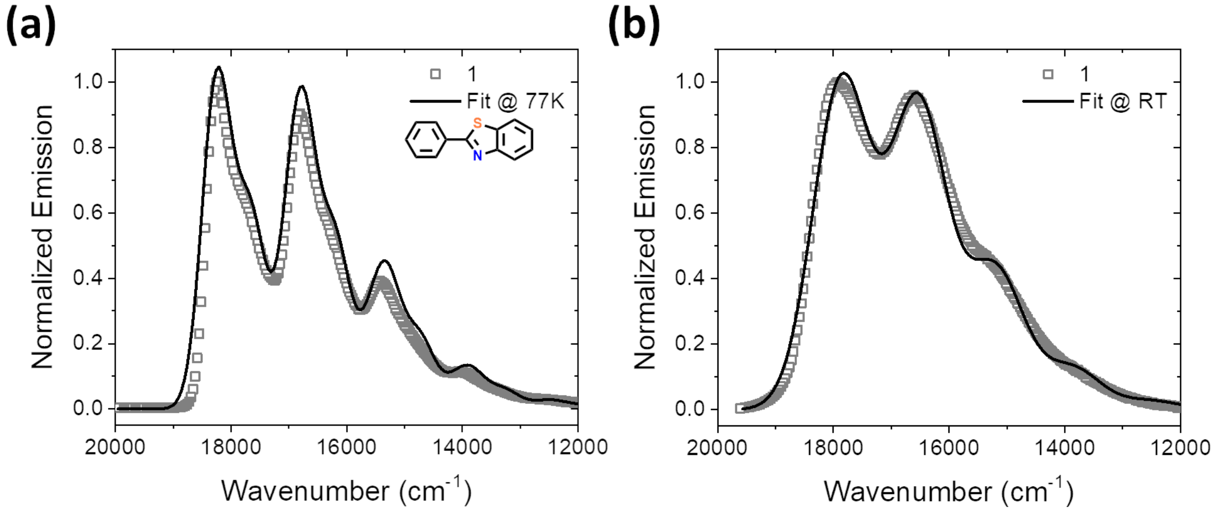

Figure S14. Emission spectra (black squares) and calculated FCLSA fit (solid line) for **1** (a) in a 2-MeTHF glass at 77 K (λ_{ex} = 450 nm) and (b) freeze-pump-thaw degassed toluene at room temperature (λ_{ex} = 500 nm).

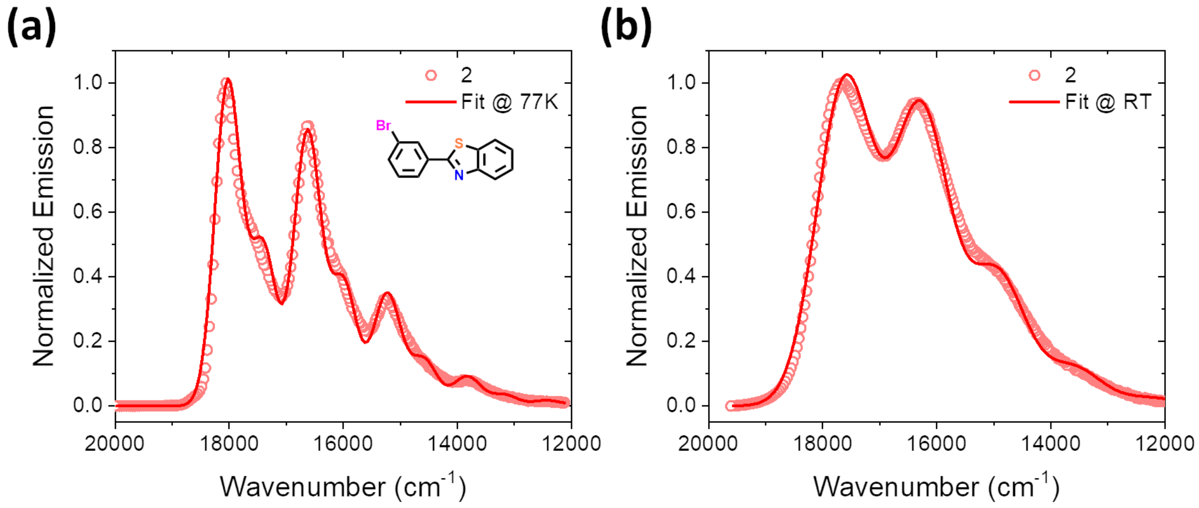

Figure S15. Emission spectra (red circles) and calculated FCLSA fit (solid line) for **2** (a) in a 2-MeTHF glass at 77 K (λ_{ex} = 450 nm) and (b) freeze-pump-thaw degassed toluene at room temperature (λ_{ex} = 500 nm).

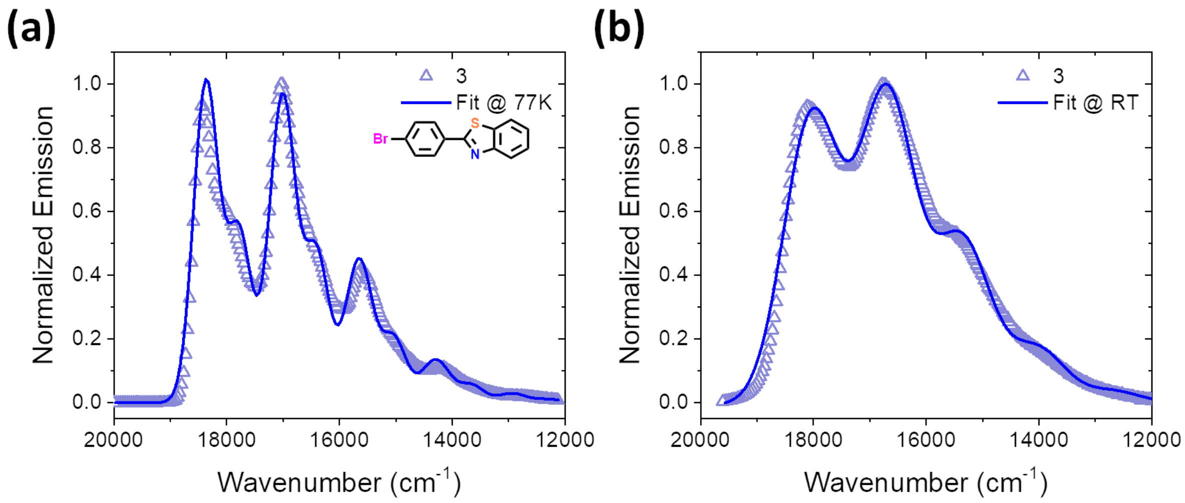

Figure S16. Emission spectra (blue triangles) and calculated FCLSA fit (solid line) for **3** (a) in a 2-MeTHF glass at 77 K (λ_{ex} = 450 nm) and (b) freeze-pump-thaw degassed toluene at room temperature (λ_{ex} = 500 nm).

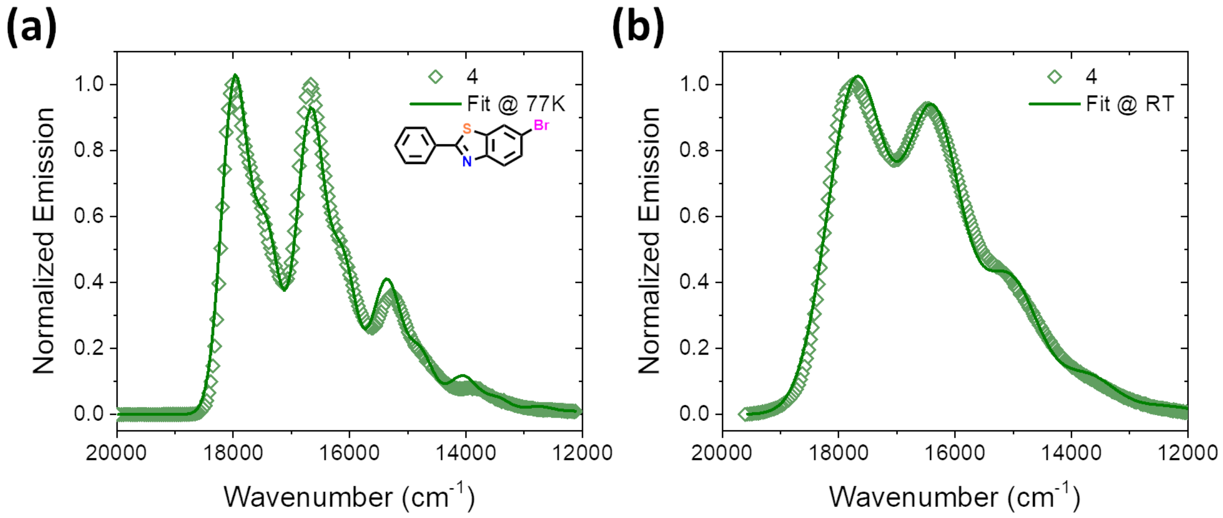

Figure S17. Emission spectra (green diamonds) and calculated FCLSA fit (solid line) for **4** (a) in a 2-MeTHF glass at 77 K (λ_{ex} = 450 nm) and (b) freeze-pump-thaw degassed toluene at room temperature (λ_{ex} = 500 nm).

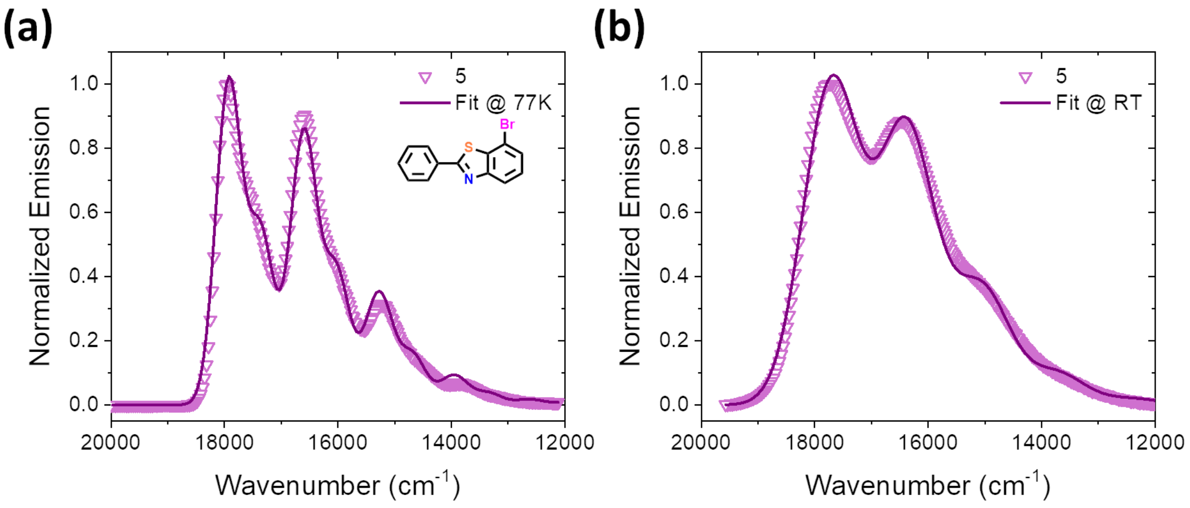

Figure S18. Emission spectra (purple triangles) and calculated FCLSA fit (solid line) for **5** (a) in a 2-MeTHF glass at 77 K (λ_{ex} = 450 nm) and (b) freeze-pump-thaw degassed toluene at room temperature (λ_{ex} = 500 nm).

Table S1. Franck-Condon line shape analysis (FCLSA) parameters calculated for complexes in a 2-MeTHF glass at 77 K (λ_{ex} = 450 nm).

Complex	E_{00} / cm^{-1}	$\Delta\nu_{1/2}$ / cm^{-1}	S_{M1}	$\hbar\omega_1$ / cm^{-1}	S_{M2}	$\hbar\omega_2$ / cm^{-1}
**1**	18248	600	1.20	1467	0.64	584
**2**	18024	537	1.05	1415	0.56	615
**3**	18368	517	1.18	1377	0.60	583
**4**	17977	526	1.11	1327	0.61	542
**5**	17927	530	1.03	1346	0.59	563

Table S2. Franck-Condon line shape analysis (FCLSA) parameters calculated for complexes in freeze-pump-thaw degassed toluene at room temperature (λ_{ex} = 500 nm).

Complex	E_0 / cm^{-1}	$\Delta\nu_{1/2}$ / cm^{-1}	S_M	$\hbar\omega$ / cm^{-1}	Chromaticity
**1**	17859	1163	1.17	1327	(0.510, 0.487)
**2**	17610	1165	1.15	1333	(0.534, 0.465)
**3**	18027	1175	1.35	1332	(0.506, 0.491)
**4**	17708	1159	1.14	1325	(0.524, 0.475)
**5**	17706	1174	1.08	1321	(0.520, 0.478)

6. Miscellaneous Photophysics

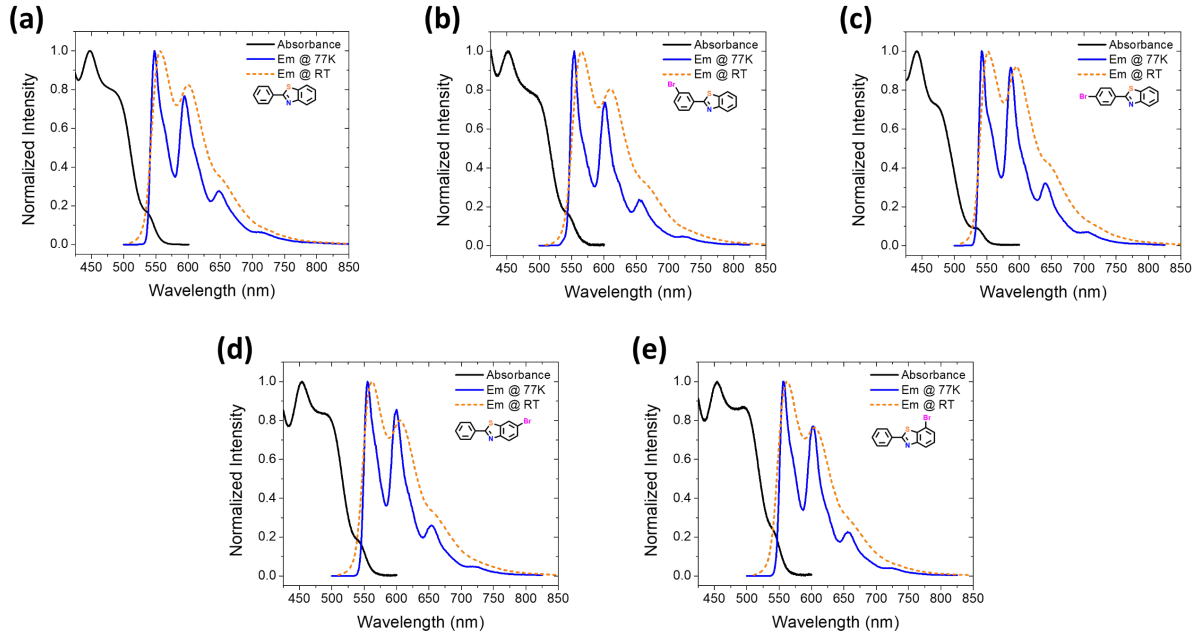

Figure S19. Absorbance (black solid line), emission at 77K (blue solid line, 2-MeTHF glass, λ_{ex} = 450 nm), and emission at room temperature, RT (orange dashed line, toluene, λ_{ex} = 500 nm) for (a) **1**; (b) **2**; (c) **3**; (d) **4**; (e) **5**.

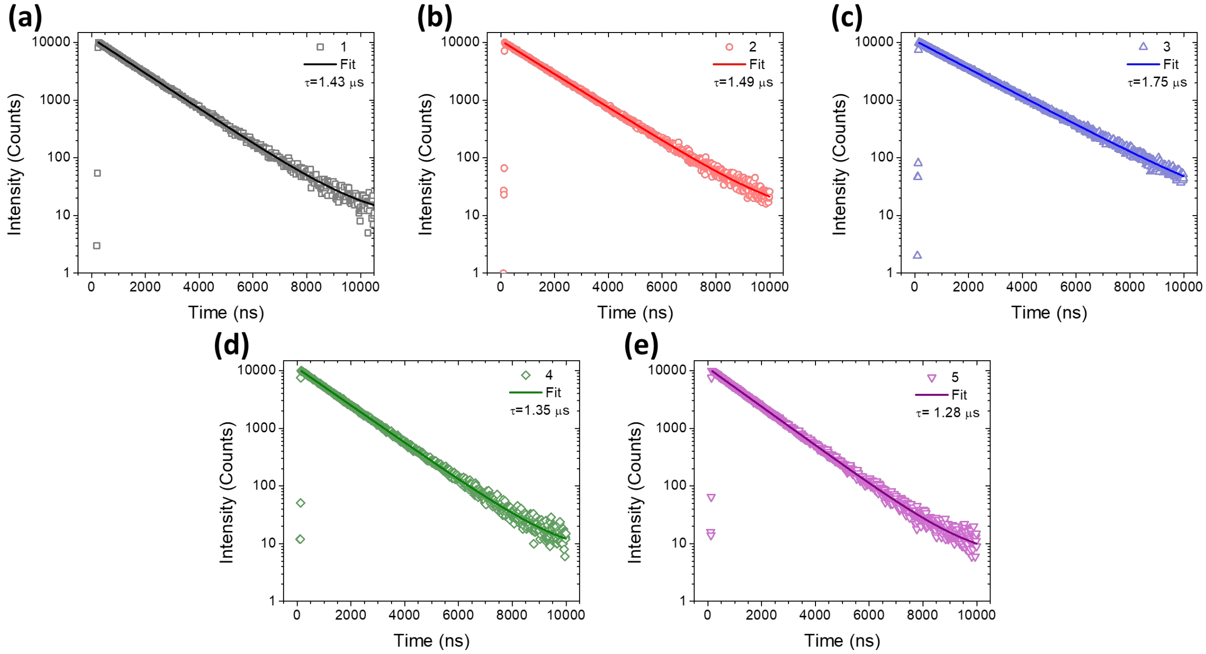

Figure S20. Emission decay traces (hollow symbols) and mono-exponential fits (solid lines) for (a) **1**; (b) **2**; (c) **3**; (d) **4**; (e) **5**. (toluene, λ_{ex} = 510 nm).

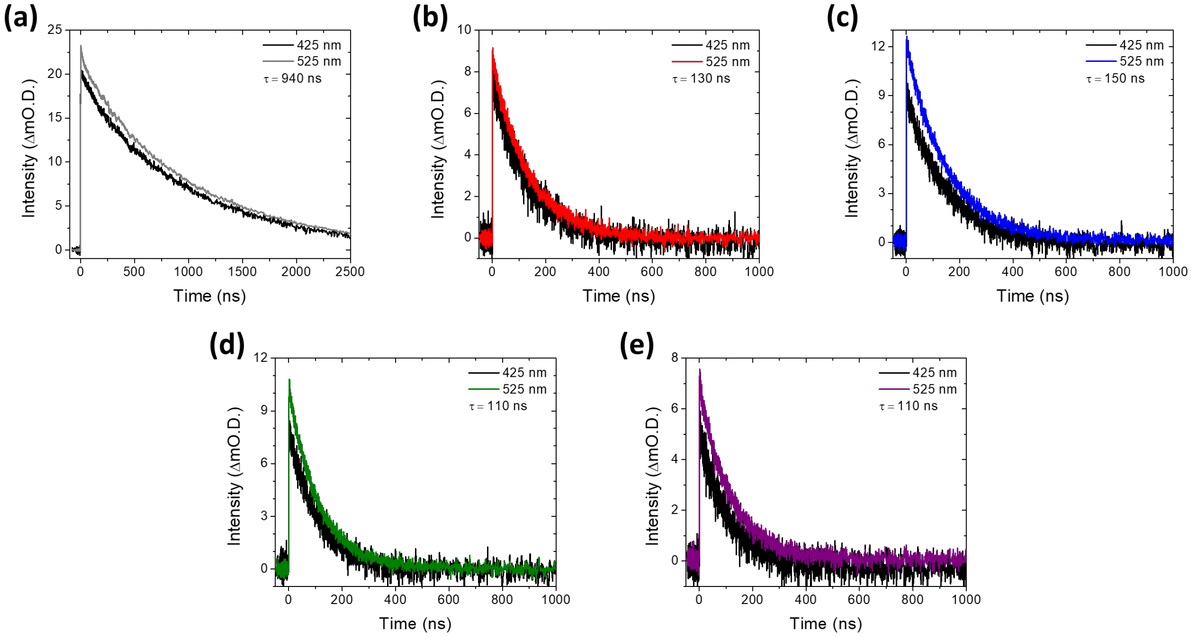

Figure S21. Decay traces obtained from nanosecond transient absorption measurements at 425 nm and 525 nm for (a) **1**; (b) **2**; (c) **3**; (d) **4**; (e) **5**. (aerated toluene, λ_{ex} = 400 nm).

Table S3. Decay lifetimes and excited state absorption peak positions for brominated complexes obtained from nanosecond transient absorption measurements (λ_{ex} = 400 nm).

Complex	λ_{ESA} / nm	$\tau_{425nm}{}^{a}$ / ns	$\tau_{525nm}{}^{a}$ / ns
**pbt**	525	942	945
**2**	530	133	133
**3**	516	151	152
**4**	543	111	110
**5**	546	111	115

[a] Monoexponential fit.

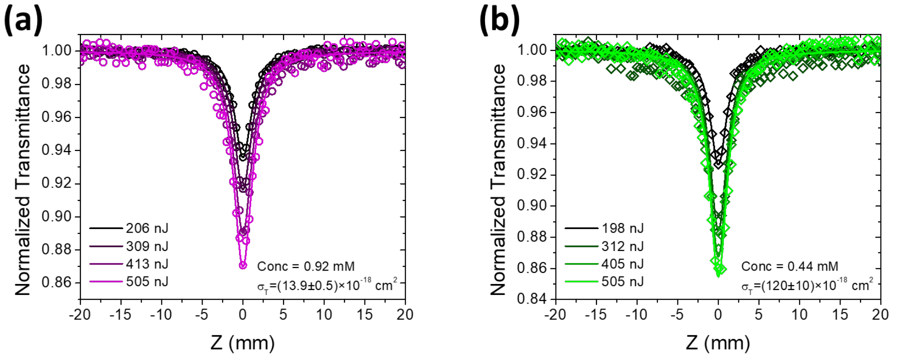

Figure S22. Open-aperture Z-scans of (a) C60 and (b) SiNc in toluene. Concentration and calculated triplet absorption cross sections are in the bottom right corner of each graph. (λ_{ex}= 532 nm).

Table S4. Fitting parameters for C60 and SiNc in toluene.

Compound	C60	SiNc
σ_g / 10^{-18} cm^2	3.2	2.6
σ_S / 10^{-18} cm^2	16	49
σ_T / 10^{-18} cm^2	13.9	120
τ_S / ns	1.25	1.5
τ_T / µs	250	290
τ_{ISC} / ns	1.25	7.1
ϕ_T	1.0	0.22

The excited-state and triplet-state cross-sections for C60 and SiNc listed in Table S4 are obtained via best fit from numerous ps and ns data collected in-house when keeping the rate constants from literature (see Section 2.5.7 of main article) fixed.

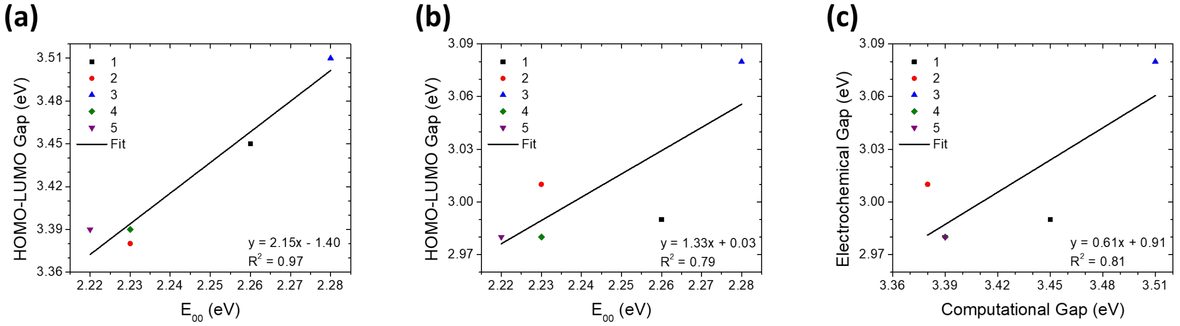

Figure S23. (a) E_{00} vs computationally-calculated HOMO-LUMO gap; (b) E_{00} vs electrochemically-calculated HOMO-LUMO gap; (c) computationally-calculated vs electrochemically-calculated HOMO-LUMO gap.

7. Computational Details:

Below is the Input Card used for all optimization and frequency calculations:

```
%CPU=128
%chk=*.chk
%mem=128GB
#b3lyp/GenECP opt SCF(XQC, maxcyc=300, maxcon=300) freq
      SCRF(Solvent=Toluene) EmpiricalDispersion=GD3

ground state optimization

0 1
Initial coordinates

Ir     0
SDD
****
Br S C H O N      0
6-311G*
****

Ir
SDD
```

Below is the Input Card used for TD DFT calculations:

```
%CPU=128
%chk=7-brpbt2.singlet.uvvis.20.chk
%mem=128GB
#b3lyp/GenECP SCRF(Solvent=Toluene) EmpiricalDispersion=GD3
TD(NStates=20)

TD DFT calculations

0 1
Optimized Coordinates

Ir     0
SDD
****
Br S C H O N      0
```

6-311G*
****

Ir
SDD

Table S6. Computational data table of corresponding to **1-5**. Compound, substituent and constitution, calculated Gibbs Free Energies, and number of imaginary frequencies in the optimized geometry.

Compound	Substituent and Constitution	Gibbs Free Energy	Number of Imaginary Frequencies
**1**	H	-2356.070236	0
**2**	3-Br	-7503.191273	0
**3**	4-Br	-7503.195886	0
**4**	6-Br	-7503.188381	0
**5**	7-Br	-7503.187640	0

Table S7. Table of the two lowest energy transitions for each complex generated by TD DFT calculations. All other transitions correspond to wavelengths shorter than 415 nm.

Compound	Excited State Number	MO # for the Transition (population)	Energy (eV)	Wavelength (nm)
**1**	1	144 ->145 (0.70)	2.6975	460
	2	144 ->146 (0.70)	2.7565	450
**2**	1	178 ->179 (0.70)	2.6617	466
	2	178 ->180 (0.70)	2.7110	457
**3**	1	178 ->179 (0.70)	2.7703	448
	2	177 ->179 (0.11)<br>178 ->180 (0.69)	2.8176	440
**4**	1	178 ->179 (0.70)	2.6531	467
	2	178 ->180 (0.70)	2.7120	457
**5**	1	178 ->179 (0.70)	2.6521	467
	2	178 ->180 (0.70)	2.7087	457

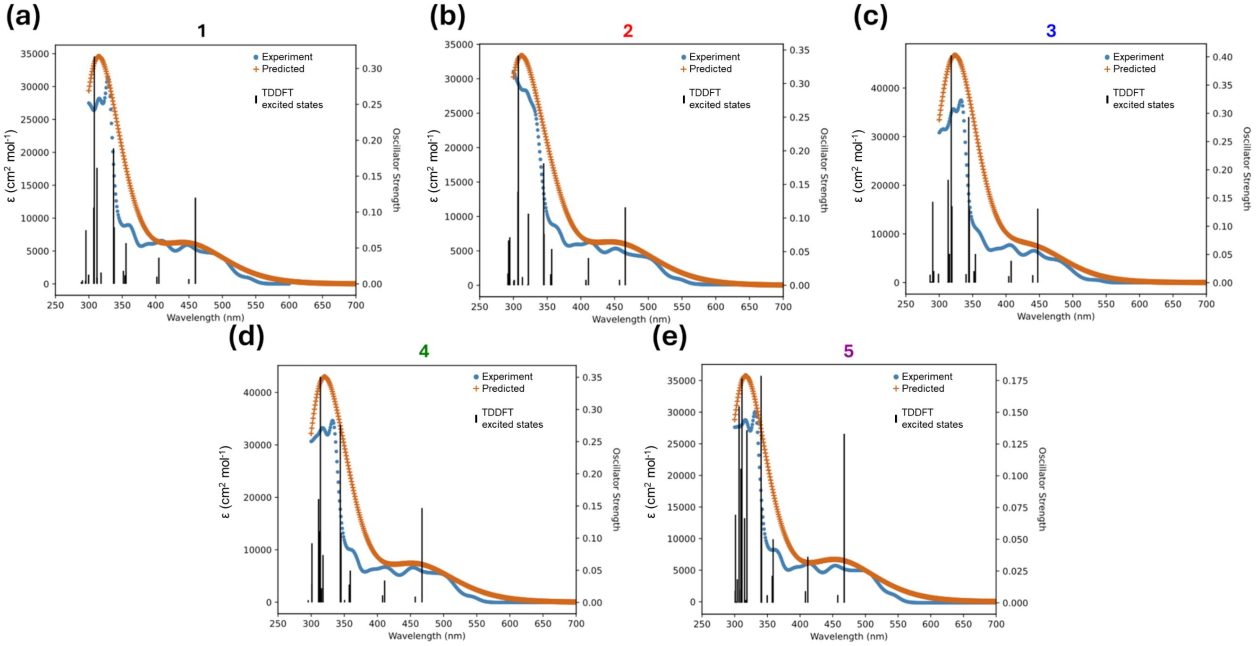

Figure S24. Experimental (blue) vs computational (orange) UV-Vis spectra for complexes (a) **1**, (b) **2**, (c) **3**, (d) **4**, and (e) **5**. TD-DFT calculated excited state peaks in black.
